# Delta1 Expression, Cell Cycle Exit, and Commitment to a Specific Secretory Fate Coincide within a Few Hours in the Mouse Intestinal Stem Cell System

**DOI:** 10.1371/journal.pone.0024484

**Published:** 2011-09-07

**Authors:** Despina Stamataki, Maxine Holder, Christine Hodgetts, Rosemary Jeffery, Emma Nye, Bradley Spencer-Dene, Douglas J. Winton, Julian Lewis

**Affiliations:** 1 Vertebrate Development Laboratory, Cancer Research United Kingdom London Research Institute, London, United Kingdom; 2 Division of Developmental Biology, National Institute for Medical Research, Mill Hill, United Kingdom; 3 Histopathology Unit Laboratory, Cancer Research United Kingdom London Research Institute, London, United Kingdom; 4 Experimental Histopathology Unit, Cancer Research United Kingdom London Research Institute, London, United Kingdom; 5 Cancer Research United Kingdom Cambridge Research Institute, Li Ka Shing Centre, Cambridge, United Kingdom; Instituto de Medicina Molecular, Portugal

## Abstract

The stem cells of the small intestine are multipotent: they give rise, via transit-amplifying cell divisions, to large numbers of columnar absorptive cells mixed with much smaller numbers of three different classes of secretory cells - mucus-secreting goblet cells, hormone-secreting enteroendocrine cells, and bactericide-secreting Paneth cells. Notch signaling is known to control commitment to a secretory fate, but why are the secretory cells such a small fraction of the population, and how does the diversity of secretory cell types arise? Using the mouse as our model organism, we find that secretory cells, and only secretory cells, pass through a phase of strong expression of the Notch ligand Delta1 (Dll1). Onset of this Dll1 expression coincides with a block to further cell division and is followed in much less than a cell cycle time by expression of Neurog3 – a marker of enteroendocrine fate – or Gfi1 – a marker of goblet or Paneth cell fate. By conditional knock-out of Dll1, we confirm that Delta-Notch signaling controls secretory commitment through lateral inhibition. We infer that cells stop dividing as they become committed to a secretory fate, while their neighbors continue dividing, explaining the final excess of absorptive over secretory cells. Our data rule out schemes in which cells first become committed to be secretory, and then diversify through subsequent cell divisions. A simple mathematical model shows how, instead, Notch signaling may simultaneously govern the commitment to be secretory and the choice between alternative modes of secretory differentiation.

## Introduction

The Notch cell-cell communication pathway [1]–[4] depends on membrane-bound receptors of the Notch family, and on membrane-bound ligands of the Delta and Jagged/Serrate families. In the phenomenon of lateral inhibition, Delta expressed in one cell binds to Notch in that cell's neighbors, triggering release of the Notch intracellular domain, NICD, into their interior, where it activates an intracellular pathway that leads to repression of *Delta*. The result is a pepper-and-salt pattern of differentiation in which some cells express Delta strongly and receive no Notch activation, while other cells receive Notch activation and do not express Delta. The different states of Notch pathway activation lead to different cell fates.

Notch signaling is thought to act in this way to drive cell diversification in the lining of the small intestine [5]–[9]. In this epithelium, a population of multipotent stem cells lying in the crypts of Lieberkuhn gives rise continually, through cell proliferation, to a mixture of terminally differentiated cell types, consisting of large numbers of columnar absorptive cells interspersed with much smaller numbers of secretory cells [10]–[15]. When Notch signaling is blocked, proliferation ceases and all cells become secretory [5], [6], [8]. Conversely, when the Notch pathway is artificially activated in all cells, proliferation is somewhat extended, and no secretory cells are produced [9]. Thus the primary fate - that of cells that escape Notch activation - is to differentiate along a secretory pathway. The secondary fate - that of cells in which Notch is activated - is either to remain as a dividing stem cell or progenitor (in the depths of the crypt, where Wnt signaling is active) or to differentiate as an absorptive cell (upon exit from the crypt and escape from the influence of Wnt) [11], [12], [16].

These findings, however, leave important questions unanswered. The differentiated absorptive and secretory cells in the gut lining are non-dividing cells generated from the multipotent stem cells through a series of transit-amplifying divisions [12], [14], [17]: how is the program of cell divisions related to the program of cell fate choices? Do any further divisions ensue, for example, after a cell has become committed to a secretory fate? The secretory cells are themselves diverse, falling into three different classes: some are mucus-secreting goblet cells, others are hormone-secreting enteroendocrine cells, and yet others are bactericide-secreting Paneth cells. When and how do cells choose between these alternative modes of secretory differentiation? What part does Notch signaling play in deciding the choices between them? And which, if any, of the known Notch ligands is responsible for mediating lateral inhibition in the gut?

In this paper, we focus on the role of one specific Notch ligand, Dll1, in the intestinal epithelium and use our findings to tackle these questions.

## Results

### Secretory cells, and only secretory cells, go through a phase of strong Dll1 expression

The standard lateral inhibition model would lead us to expect that cells becoming committed to a secretory fate (because they escape Notch activation) should strongly express one or more Notch ligands, while those heading for an absorptive fate (because they are subject to Notch activation) should not. To test whether the Notch ligand Dll1 behaves in this way in the gut, we examined its pattern of expression using a line of mice with *lacZ* inserted at the *Dll1* locus, producing β-galactosidase (β-gal) as a reporter for *Dll1* [18]. Homozygotes die as embryos, but the *Dll1^lacZ/+^* heterozygotes are healthy and fertile. Because β-galactosidase protein has a long half-life, of approximately two days [19], [20], β-galactosidase staining marks cells that have expressed *Dll1* in the past as well as those expressing it currently.

**Figure 1:**
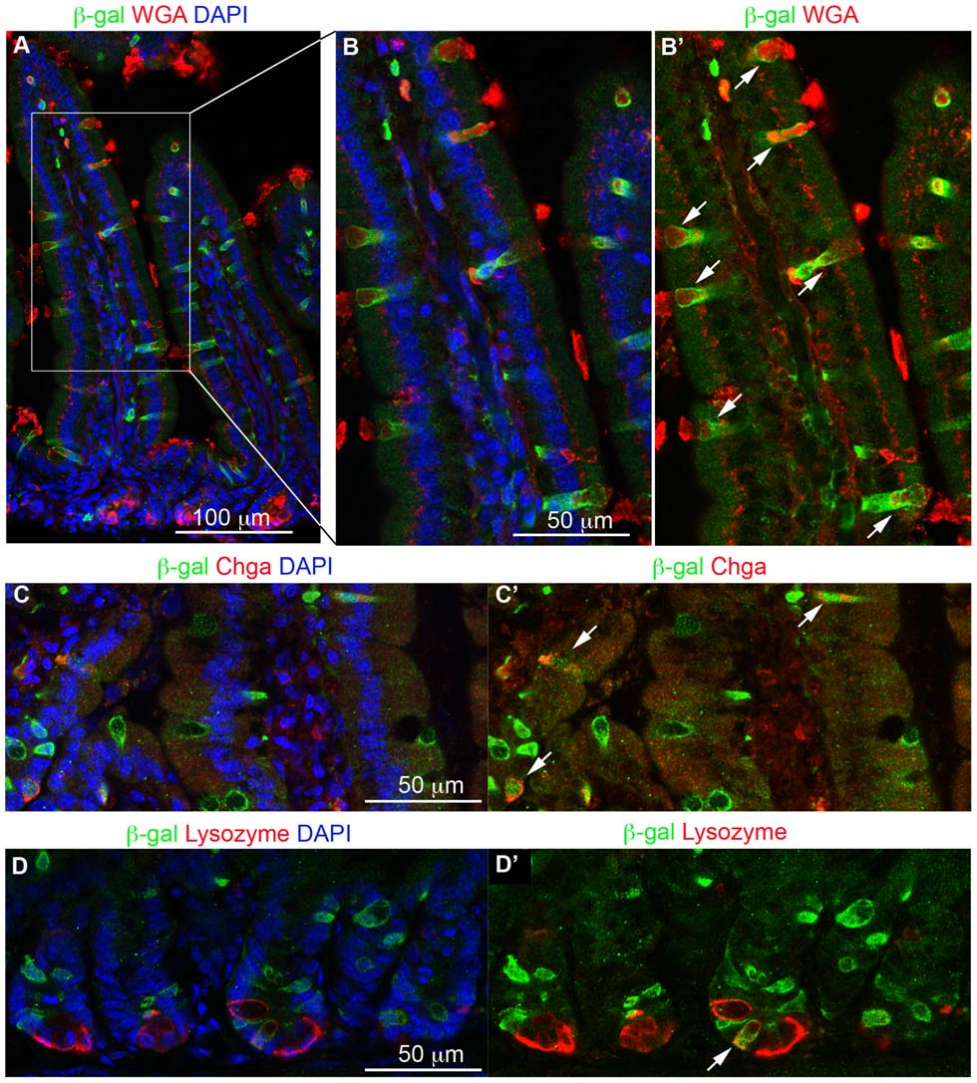
All three classes of secretory cells express the *Dll1^lacZ^* reporter. (**A,B,B′**) β-galactosidase immunostaining (green) is seen in practically all cells stained for the goblet-cell marker wheat-germ agglutinin (WGA). B, B′ are enlargments of the boxed area in A. (**C, C′**) β-galactosidase immunostaining (green) is seen in many of the cells stained for the general enteroendocrine marker chromogranin A (Chga, red). (**D, D′**) β-galactosidase immunostaining (green) is seen in occasional Paneth cells, identified by staining for lysozyme. Arrows point to cells where β-galactosidase co-localizes with the corresponding marker. DAPI staining for DNA in blue. All pictures show proximal small intestine of adult *Dll1^+/lacZ^* mice.

β-galactosidase-positive cells were scattered throughout the intestinal epithelium of the *Dll1^lacZ/+^* mice and could be categorized by appropriate double staining. We used wheat-germ agglutinin (WGA) staining to identify goblet cells, and immunostaining for chromogranin A (Chga) as a general marker for enteroendocrine cells. In a survey of the epithelium, WGA-positive cells represented 6.7±0.8% of the total epithelial population, and the Chga-positive cells 1.2±0.1% (mean ± s.e.m., n = 3 mice, >2000 cells scored). Of the WGA-positive cells, almost all (96%, out of 161 WGA-positive cells counted in a set of sample fields from two mice) were β-galactosidase-positive (Figure 1A,B,B′). Of the Chga-positive cells, 66% (out of 131 Chga-positive cells counted in a set of sample fields from three mice) were β-galactosidase-positive (Figure 1C,C′). We verified the β-galactosidase-positivity of enteroendocrine cells by immunostaining also for the gut hormones serotonin, somatostatin, glucagon and ghrelin (data not shown): each of these was also seen in some β-galactosidase-positive cells. The third class of secretory cells in the small intestine, the Paneth cells, only rarely showed β-galactosidase staining (Figure 1D,D′). We never saw any β-galactosidase staining in the absorptive cells, even though these constitute more than 90% of the epithelial population.

**Figure 2:**
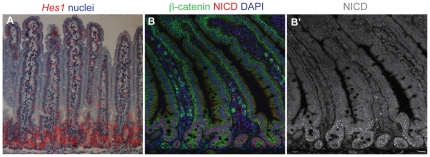
The Notch signaling pathway is activated in the crypts. (**A**) In situ hybridisation showing expression of *Hes1* mRNA (red silver grains; radioactive probe detection, false color). (**B**) Immunofluorescence staining for NICD (red), with β-catenin in green and DAPI in blue. (**B′**) shows the NICD channel only. Note that NICD and *Hes1* mRNA are largely confined to the crypts, implying that the crypts are the site of Delta-Notch signaling. The nuclear dots of NICD immunostaining resemble those seen in other studies [55], [56]; a caveat is that this staining may reveal NICD lingering in degradation bodies and not purely the NICD that is active as a transcription factor. Perdurance of NICD immunoreactivity may explain why we saw nuclear NICD staining in a substantial proportion of crypt secretory cells as well as in their non-secretory neighbours (data not shown).

These findings indicate that secretory cells, and only secretory cells, indeed go through a phase of strong Dll1 expression at some point in their developmental history; and this expression must be early and transient, since in situ hybridisation reveals that *Dll1* mRNA is restricted to scattered cells that are confined to the crypts [21], [22]. The intracellular active fragment of Notch, NICD, and the mRNA product of the Notch target gene *Hes1* (see also [7], [22]) are both also largely confined to the crypts, with expression fading to zero as cells emerge onto the bases of the villi (Figure 2A,B,B′)). Evidently, it is only in the crypts that cells interact via the Notch pathway, and only there that Dll1 can be serving to activate Notch.

The restriction of Dll1 expression to the region where new cells are born explains why, although many enteroendocrine cells stained for β-galactosidase, some did not: their relatively long dwell time in the epithelium (4.0 days (in jejunum) as opposed to 2.3–2.9 days for the goblet cells [10]), allows time for disappearance of β-galactosidase protein following transient expression of Dll1. The same applies even more strongly to Paneth cells, which are estimated to persist for 57 days [23]: if β-galactosidase perdures in them for 2–3 days following determination, one would expect to see only about 5% of them labeled with β-galactosidase; and this is consistent with our observations.

### Dll4 is coexpressed with Dll1 in secretory cells

**Figure 3:**
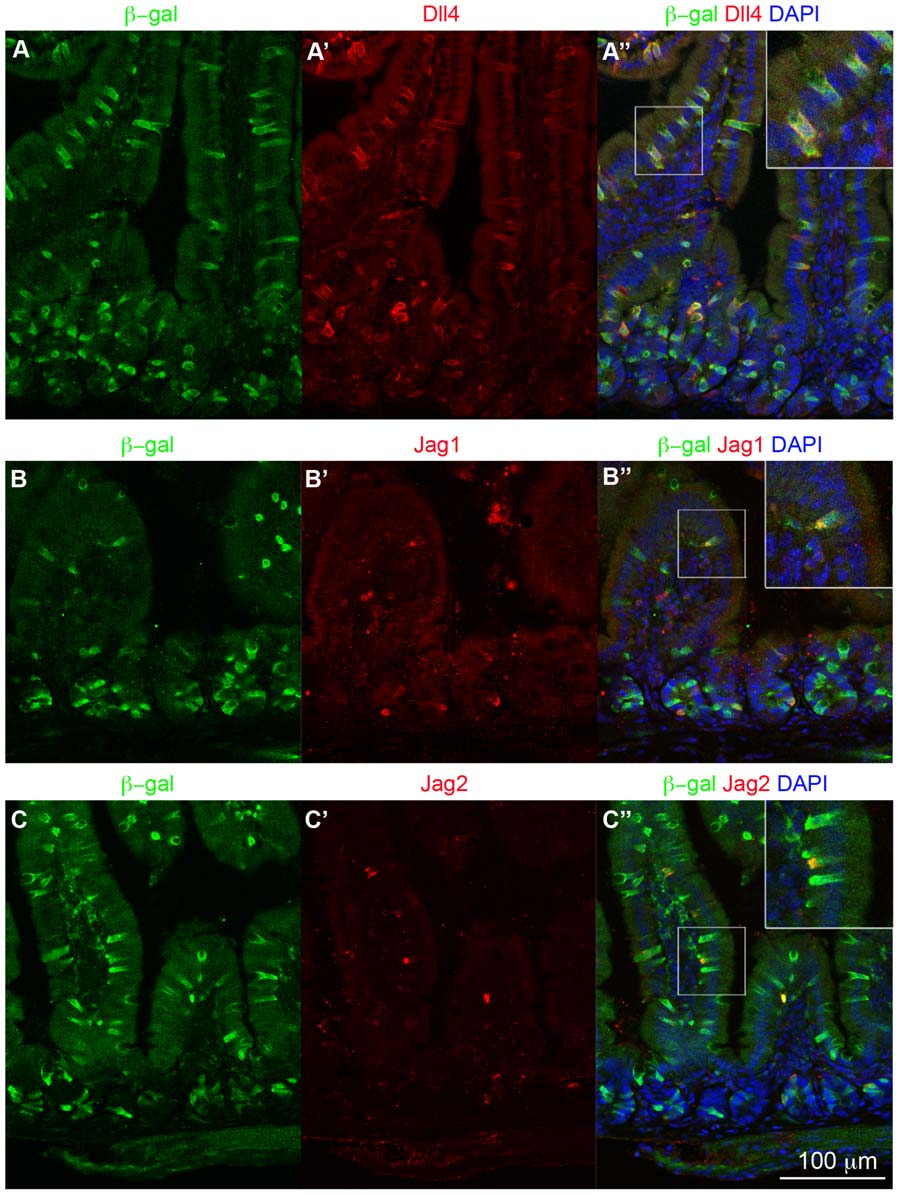
Other Notch ligands are co-expressed with Dll1 in proximal small intestine. (**A–C**) Immunofluorescence staining for Dll4 (A-A″), Jag1 (B-B″) and Jag2 (C-C″) (red) on cryosections of small intestine from *Dll1^lacZ/+^* mice; β-galactosidase immunostaining in green and DAPI staining for DNA in blue. Insets in A″, B″ and C″ are enlargements of the adjacent boxed areas, showing co-expression of these other Notch ligands with the β-galactosidase Dll1 reporter.

Other Notch ligands besides Dll1 are also expressed in the gut [22], [24], and we used immunostaining to examine their distribution (Figure 3; for Dll1 itself we have no satisfactory antibody). A Dll4 antibody stained many, if not all, of the secretory cells, and this staining coincided with immunostaining for β-galactosidase in the *Dll1^lacZ/+^* mice (Figure 3A-A″), implying that Dll1 and Dll4 are expressed in the same cells and thus may function quasi-redundantly in this context (see below). Antibodies against two other Notch ligands, Jag1 and Jag2, stained a few sparsely scattered cells, which were also β-galactosidase-positive and were located both in the villus epithelium and in the crypts (Figure 3B-B″, C-C″).

### *Dll1* knockout leads to increased numbers of secretory cells

**Figure 4:**
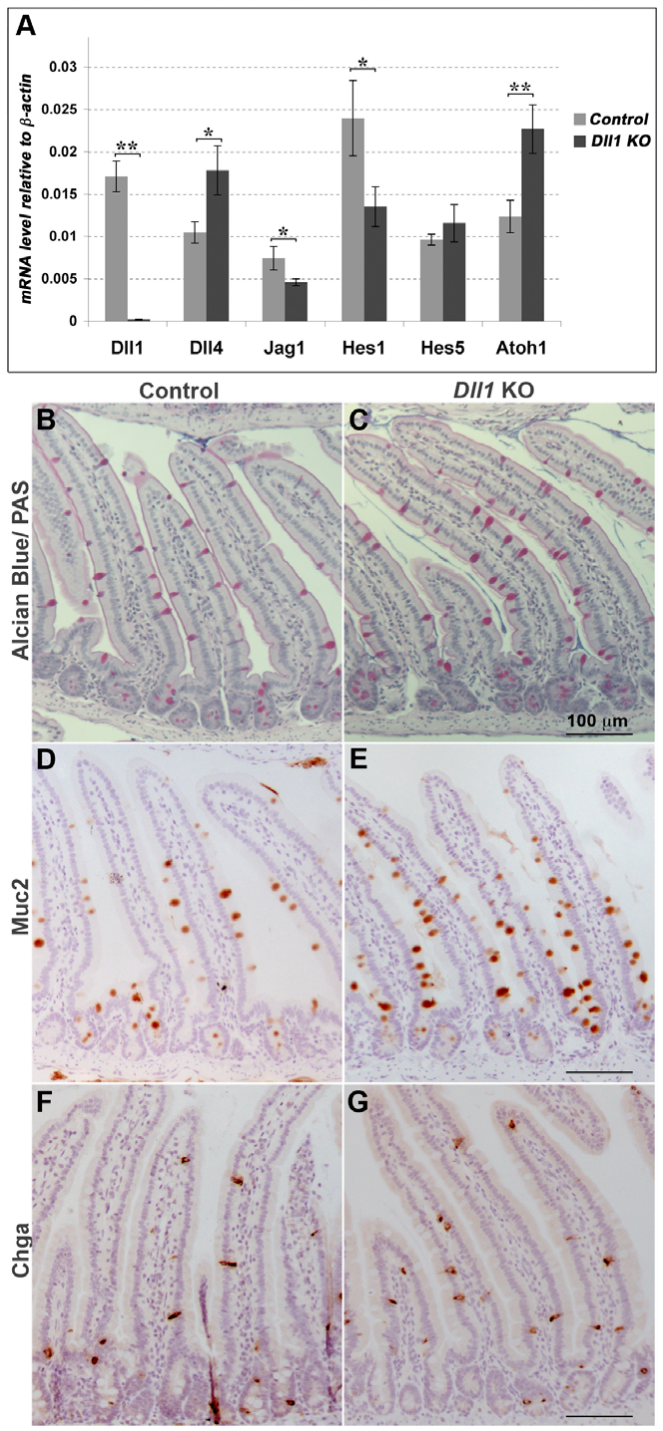
Loss of *Dll1* reduces lateral inhibition and Notch pathway activation and increases secretory cell numbers. (**A**) qRT-PCR analysis of levels of expression of Notch pathway components in isolated epithelium of proximal small intestine after conditional knock-out of *Dll1*. The knockout data (Dll1 KO) are from *Dll1^flox/flox^;AhCre* mice killed 12 days after the initial treatment with the inducer β-naphthoflavone. Control mice were *Dll1^flox/flox^* lacking the *AhCre* transgene, but similarly injected with β-naphthoflavone. Reduced expression of *Hes1* and increased expression of *Dll4* and *Atoh1* reflect a reduction in Notch signaling activity and in lateral inhibition. Error bars show standard errors of the means of measurements from 5 knockout and 6 control mice. * and ** denote statistically significant effects (one-tailed t-test; p = 0.05 for *Jag1*, p = 0.04 for *Hes1*, p = 0.02 for *Dll4*, p = 0.007 for *Atoh1* and p<10^−5^ for *Dll1*). We were unable to quantify *Jag2* reliably in our samples. (**B, C**) Alcian blue/periodic-acid-Schiff (PAS) staining shows more goblet cells in the *Dll1* conditional knockout (C) than in control (B) mice. (**D, E**) Mucin2 (Muc2) immunostaining of goblet cells shows the same phenomenon. (**F,G**) Chromogranin A (Chga) immunostaining, identifying enteroendocrine cells in control (F) and *Dll1* conditional knockout (G) mice.

To check that Dll1 is a regulator, and not merely a marker, of cell fate choices in the intestinal epithelium, we knocked out *Dll1* function acutely in the intestinal epithelium of adult mice that were homozygous for a floxed *Dll1* allele [25], [26] and contained the *AhCre* transgene, which expresses Cre in the intestine in response to β-naphthoflavone [27]. Mice received intraperitoneal injections of β-naphthoflavone on three consecutive days and were killed for analysis 5, 12 or 28 days after the first injection. They appeared healthy up to this time and their body weight was not altered significantly. Quantitative RT-PCR (qRT-PCR) analysis confirmed that recombination had occurred, inactivating the *Dll1* gene, in almost all cells of the intestinal epithelium (Figure 4A).

Focusing first on the knockout mice killed at 12 days after injection, and comparing them with controls similarly injected with β-naphthoflavone but lacking *AhCre*, we found an 84% increase in the proportion of goblet cells (Figure 4B–E) (goblet cells/total villus cells = 0.123±0.005 in the knockout versus 0.067±0.008 in the control; mean ± s.e.m., n = 3 mice for each condition, >2000 cells scored for each mouse). The proportion of Chga-positive (enteroendocrine) cells (Figure 4F,G) was increased even more, by 148% (Chga^+^ cells/total villus cells = 0.030±0.002 in the knockout versus 0.012±0.001 in the control; mean ± s.e.m., n = 3 mice for each condition, >2500 cells scored for each mouse). Paneth cell numbers, as indicated by lysozyme immunostaining (not shown), seemed unaffected by loss of Dll1, but their long lifetime means that their numbers will not have had time to adjust noticeably over the course of the 12-day experiment.

The qRT-PCR measurements (Figure 4A) showed that the changes in secretory cell numbers went with changes in Notch pathway gene expression: expression of *Dll4* was increased by 70%, that of *Hes1* was decreased by 44%, and that of *Atoh1* - a sign of commitment to a secretory fate [28], [29] - was increased by 84%. These effects are all in accord with standard expectations for a system in which Dll1 delivers lateral inhibition via Notch to restrict commitment to a secretory fate, with *Hes1* and the *Delta* genes as main mediators of inhibition. Changes in expression of *Hes5* and *Jag1* followed a different pattern (Figure 4A), suggesting that these components are regulated differently, as in some other systems [30]; indeed, other studies show that in the intestine *Hes5* is negatively regulated by *Hes1* [7] and appears to have an opposite influence on secretory differentiation [31].

Results for mice fixed earlier or later, at 5 or 28 days after the first injection, were similar. At 5 days, goblet cell numbers in the knockout were already increased, by 105% (goblet cells/total villus cells = 0.122±0.010 in the knockouts versus 0.060±0.006 in the controls; >4000 cells scored for each mouse, n = 2 mice). In mice fixed 28 days after the first injection, there was still a strong (65%) increase in goblet cell numbers (goblet cells/total villus cells = 0.116±0.002 in the knockouts versus 0.071±0.005 in the controls; mean ± s.e.m., >1300 cells scored for each mouse, n = 3 mice for each condition). The apparent change in the effect on goblet cell numbers from 5 to 12 to 28 days after injection may be statistically significant but is not likely to be due to change in the surviving proportion of recombined cells: *Dll1* mRNA levels in the knockout mice at the 28 day time point remained less than 4% of the controls, as measured by qRT-PCR (not shown). We also counted Paneth cells in our 28 day specimens: at this later time point we did see an increase, by about 30% (3.8±0.4 lysozyme-positive cells per crypt section in the knockout mice, versus 2.9±0.2 in the littermate controls; mean ± s.e.m. of 3 mice for each condition, >12 crypts counted in each mouse; significantly different at the p = 0.03 level by t-test).

### Dll1 and Dll4 are joint regulators of commitment to a secretory fate

The effects of knocking out *Dll1*, as described above, are similar to the effects of a complete failure of Notch signaling [5], [6], [8], although milder, as one would expect given that Dll1 is only one of several Notch ligands expressed in the secretory cell lineage. In both cases, cells are diverted from an absorptive to a secretory fate. Dll1 clearly regulates the choice of pathway of differentiation, and evidently does this by preventing the neighbors of a *Dll1*-expressing cell from becoming secretory. Escape from such inhibition permits expression of *Dll1* and entails a secretory fate.

These conclusions are reinforced by the findings of a study conducted in parallel with the present work, investigating mice in which both *Dll1* and *Dll4* have been conditionally deleted in the intestine [32]. As noted above, we have shown that these two Notch ligands are co-expressed in the developing secretory cells. When both genes are knocked out, practically all the intestinal cells are converted to a secretory fate. This resembles the effect of other manipulations that block Notch signaling totally [5], [6], [8], and it confirms that Dll1 and Dll4, expressed in the same cells and acting in parallel in quasi-redundant fashion, are the key Notch ligands regulating commitment to a secretory fate in the small intestine.

### Expression of Dll1 in each future secretory cell goes hand-in-hand with withdrawal from the cell cycle

**Figure 5:**
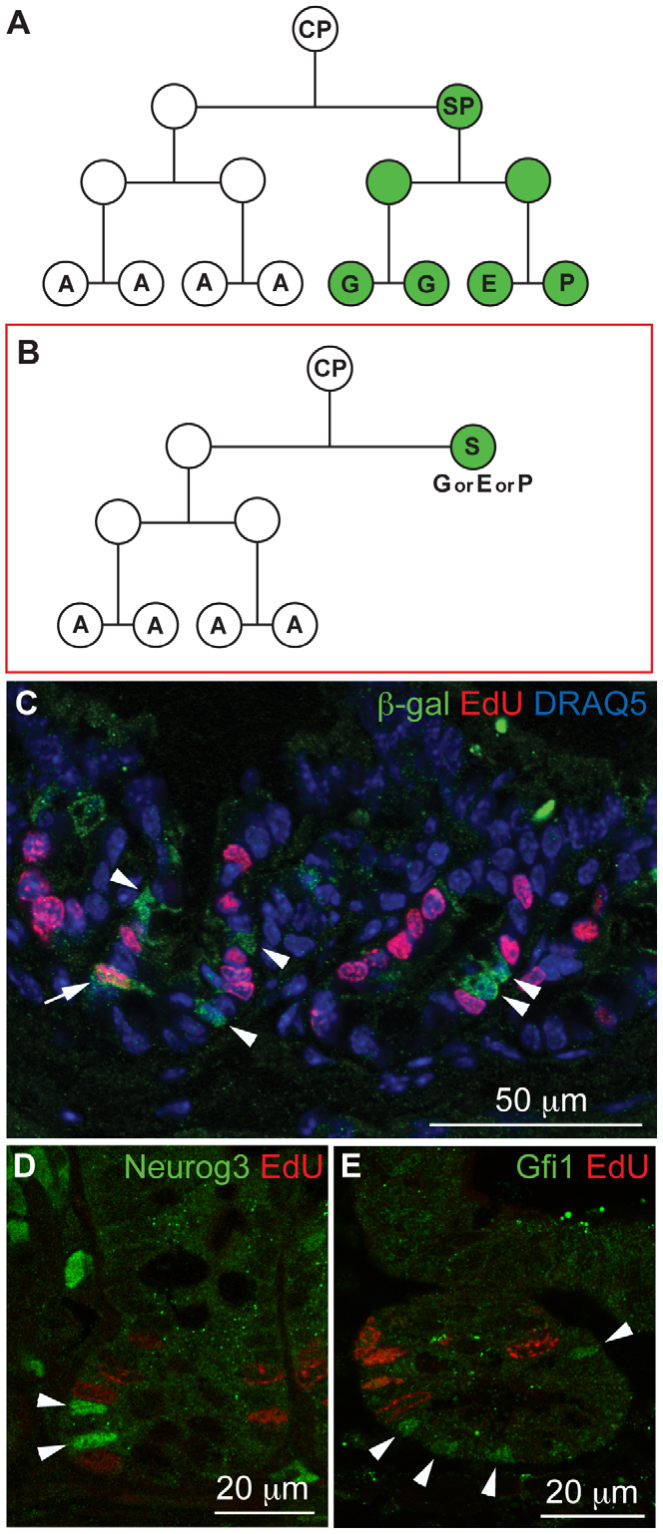
Dll1-expressing crypt cells are almost all postmitotic. (**A, B**) Two possible models of the intestinal cell lineage tree. Our data favour the second model (B). (CP - common progenitor; SP - secretory progenitor; A - absorptive cell; S - secretory cell; G - goblet cell; E - enteroendocrine cell; P - Paneth cell). (**C**) Immunofluorescence staining for β-galactosidase protein (green) combined with staining for EdU incorporation (red) in crypts of *Dll1^+/lacZ^* mice, after a 1-hour pulse of EdU, with DRAQ5 nuclear staining in blue; almost all (93%) of the β-galactosidase-positive cells are EdU-negative. Arrowheads point to β-galactosidase^+^ EdU^−^ cells; arrow points to a rare β-galactosidase^+^ EdU^+^ cell. (**D, E**) Immunofluorescence staining for the secretory cell markers Neurog3 (D, green) and Gfi1 (E, green), combined with staining for EdU incorporation (red). Even fewer of the cells expressing these markers of secretory specialization stain positive for EdU incorporation. Arrowheads point to nuclei positive for the secretory markers.

These findings leave open an important question: at what point in the history of the secretory cell does commitment happen? Does the escape from Notch activation, accompanied by the rise in Dll1 expression, occur in a progenitor that then continues to divide, giving rise to a clone of many secretory cells (Figure 5A)? Or does it go with cessation of cell division, committing only a single cell to be secretory (Figure 5B)?

To find out, we combined immunostaining for β-galactosidase with staining for EdU incorporation to identify cells in S phase [33], in *Dll1^lacZ/+^* mice that had been killed one hour after an EdU injection (Figure 5C). Only 7.0±2.5% of the β-galactosidase-stained cells in the crypts were EdU-positive, as against 38±3% EdU-positivity for the β-galactosidase-negative crypt cells (mean ± s.d., n = 5 mice, total of 5318 crypt cells counted). Thus, while a small minority of Dll1-expressing cells are found in S phase and are therefore destined to divide, it appears that most, if not all, of these cells have withdrawn from the cell cycle. This implies that the future secretory cells withdraw from the cell cycle at approximately the same time at which they express Dll1 strongly - which is to say, at the time at which they become committed to a secretory fate.

In general signals governing cell proliferation act by controlling passage past a certain “Start” or “Restriction point” in the cell cycle, usually located in the G1/G0 phase of the cycle, some time before the onset of S phase (and necessarily no later). A cell that has already passed this point will go on to complete the current cycle regardless of any signal to stop proliferation. Thus even if cells experience such a stop signal as soon as they begin to express Dll1, it is inevitable that some of them will nevertheless be found in S phase because they have already passed Start. As explained in detail in Text S1, the fraction of Dll1-expressing cells in the crypt predicted to be found in S phase for this reason depends on the delay, if any, from onset of Dll1 expression to onset of the stop signal, and on the cell-cycle and population-kinetic parameters for the intestinal crypt, which are well documented [34]. Through this argument, using the available data and given the observed fraction of Dll1-expressing cells that are found in S-phase, we can deduce the timing of the stop signal relative to onset of Dll expression.

**Figure 6:**
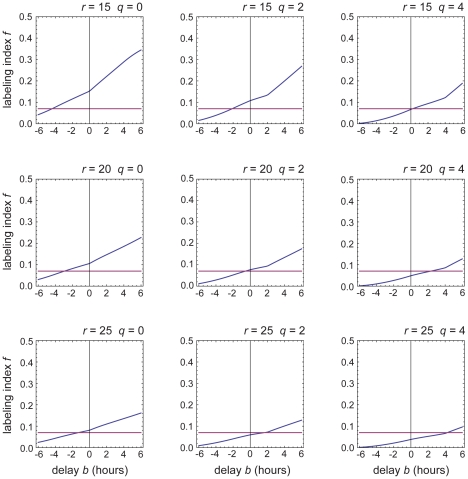
Onset of Dll1 expression and onset of the block to cell cycling must be coincident within ±4 hours. The graphs show the predicted S-phase labeling index (blue curve) for cells that have switched on Dll1 (as indicated by the β-galactosidase reporter) as a function of the delay *b* between this event and onset of the cell-cycle block. Results are shown for each of a range of possible values of the cells' average crypt residence time *r* (taken to be somewhere between 15 and 25 hours [34]) and of the timing *q* of Start in the cell cycle (taken to lie somewhere between the beginning of G1 phase (*q* = 0) and the beginning of S phase (*q* = 4 hours [34])). The horizontal purple line marks the measured value of the labeling index for cells expressing the Dll1 reporter. From the point of intersection of the line and the curve, we can read off the value of the delay that must be postulated to explain the observed labeling index on the assumption of the specified values of *r* and *q*. No matter what values we assume for *r* and *q* within these plausible ranges, we see that the delay *b* cannot be more than ±4 hours. See Text S1 for full details.

A rigorous quantitative analysis is provided as Text S1. Figure 6 shows the results: for the experimental data to match the predictions, we find that the signal for arrest of cell cycling must come into force somewhere between 4 hours before, and 4 hours after, the cell switches on high-level Dll1 expression as indicated by the β-galactosidase reporter.

### Markers specific for goblet or enteroendocrine cells begin to be expressed about three to five hours after the onset of Dll1 expression

It is nevertheless conceivable that some small proportion of Dll1-expressing cells might continue to divide for further cycles. As a further check on our conclusions, therefore, we examined the EdU labeling index, measured as above, for cells in the crypts expressing other early markers of commitment to a secretory fate. Gfi1 expression is a marker of goblet and Paneth cell fate [35], [36], while Neurog3 expression is a marker of enteroendocrine fate [36]–[38]. Both proteins are transcription factors that operate early in secretory development to dictate the choice of secretory subtype, with Gfi1 serving to stabilise repression of Neurog3 in the future goblet and Paneth cells [36].

Immunostaining for Gfi1 in mice pulse-labeled as above with EdU showed that only 1.4% of Gfi1-positive nuclei in the crypt were EdU-positive (Figure 5E; n = 370 Gfi1-positive cells counted, from 134 crypts, 5 mice). Immunostaining for Neurog3 showed that 3.6% of Neurog3-positive nuclei were EdU-positive (Figure 5D; n = 194 Neurog3-positive cells counted, from 137 crypts, 3 mice). These counts reinforce the conclusion that cells stop dividing as they become committed to a secretory fate, and they indicate that Dll1 expression, as manifest in β-galactosidase staining, slightly precedes expression of Gfi1 or Neurog3. In fact, from the calculations in Text S1, we can estimate that, to account for the differences of labeling index, onset of Neurog3 expression and of Gfi1 expression must be later than onset of Dll1 expression by roughly 3 hours and 5 hours, respectively.

## Discussion

Our results add to the already strong evidence that Notch signaling controls commitment to a secretory fate in the intestine, and they extend our understanding of this process in several respects. We identify Dll1 as a key Notch ligand in the intestine and show that it is co-expressed with Dll4, suggesting that the two ligands may function quasi-redundantly to mediate lateral inhibition – a suggestion that is confirmed in a parallel publication [32]. On this basis, we demonstrate that the onset of strong Dll1 expression in a cell marks the point of its commitment to a secretory fate and goes hand-in-hand with withdrawal from the cell division cycle. Our quantitative analysis implies that the block to cell cycling comes into force at the onset of Dll1 expression (as detected by our β-galactosidase reporter), within ±4 hours. Moreover, from our measurements of labeling index we are able to estimate that the future secretory cells become specialised as goblet/Paneth (expressing Gfi1) or enteroendocrine (expressing Neurog3) within five hours (much less than the cell cycle time, which is ∼13 hours [34]) after the onset of Dll1 expression.

### Future secretory cells cease dividing at the time of their commitment to a secretory fate

A common suggestion in the published literature is that cells continue to divide after commitment to a secretory fate and diversify through these subsequent divisions [11], [28], [39]. It is hard to find any clear assertion of our contrary view; yet the available data, though scanty, are largely consistent with it. Thus Bjerknes and Cheng [37], analysing genetically marked clones, report that they never saw the combinations of different secretory cell types that would be expected on this hypothesis of division-coupled secretory diversification, whereas they frequently saw enteroendocrine cells originating as non-dividing sisters of future absorptive cells. The proposition that commitment to a secretory fate and cessation of cycling are linked consequences of an escape from Notch-mediated lateral inhibition fits a further finding: when *Notch1* and *Notch2* are knocked out acutely in the intestine, the cells switch on expression of the cyclin-dependent kinase inhibitors p27^Kip1^ and p57^Kip2^ and stop dividing [6]. This effect of Notch signaling on cell division appears to be mediated by Atoh1, a bHLH transcriptional activator whose expression is necessary [28], [40] and sufficient [41] to commit gut cells to a secretory fate: when a cell escapes from Notch activation, it switches on expression of Atoh1, and this is required both to halt proliferation and to drive differentiation [29], [42], [43].

### The regulation of commitment and cell division by Delta-Notch signaling accounts for the normal ratio of absorptive to secretory cells

**Figure 7:**
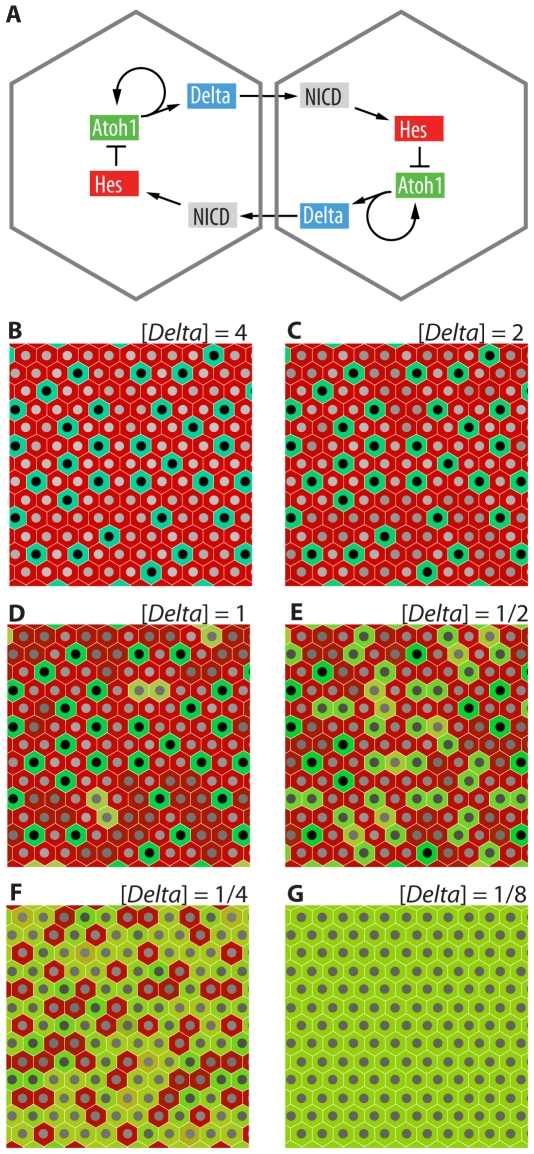
Mathematical modeling predicts increased numbers of secretory cells at lower *Delta* gene dosages. (**A**) The postulated gene-regulatory circuitry of lateral inhibition in the gut epithelium, depicted for a pair of adjacent interacting cells. (**B–G**) The computed steady-state pattern that emerges when an array of many cells interact with one another in this way, starting from a condition of randomly varying low-level expression of the genes in each cell. Each panel shows the outcome for a different effective dosage of *Delta* genes; gene dosage [*Delta*] = 1 is taken to represent wild-type. Lower values represent effects of knocking out *Dll1* while retaining some *Delta* gene function due to other *Delta* or *Jagged* genes that remain intact. Colors represent levels of gene products in each cell - green for *Atoh1*, blue for *Delta*, red for *Hes*, and composite colors for mixtures. The brightness of the nucleus represents the level of NICD - white high, black low. High values of [*Delta*] lead to a binary outcome, where each cell settles into one or other of just two possible states. Very low values of [*Delta*] lead to uniform strong expression of *Atoh1*. Intermediate values of [*Delta*] give a richer pattern, in which some cells that express *Atoh1* occur in isolation (and appear green) while others are contiguous and have somewhat lower levels of *Atoh1* (and appear yellow) because they activate Notch in one another and consequently also express *Hes*. We suggest that the higher, lower, and zero levels of *Atoh1* could correspond to goblet, enteroendocrine, and absorptive cell fates, respectively.

Our findings provide a straightforward explanation for the ratio of secretory to absorptive cells - about 1∶13 in the proximal small intestine. Simple models of lateral inhibition typically generate a 1∶3 ratio of primary-fate (Delta-expressing, secretory) cells to secondary-fate (Notch-activated) cells [44] - see Figure 7. Thus the machinery of Notch-mediated lateral inhibition, followed by about two extra divisions in the Notch-activated cells (destined for an absorptive fate, and still capable of dividing so long as they remain under the influence of Wnt in the crypt), is sufficient to account for the normal observed secretory∶absorptive ratio. This fits with previous work showing that in the mouse small intestine those cells that continue to proliferate undergo an average of just over two cell divisions on their way through the transit-amplifying compartment [34].

### A mathematical model of Delta-Notch signaling predicts an altered ratio of cell types at reduced Delta gene dosage

Does Delta-Notch lateral-inhibition theory satisfactorily explain the change in the secretory∶absorptive ratio when we knock out *Dll1*? A reasonable proposal for the lateral inhibition circuitry, based on published data [11], [28], [29], [45], [46], is as shown in Figure 7A. According to this scheme, cells that are initially equivalent, expressing Delta at similar levels, deliver inhibitory signals to one another, tending to reduce Delta expression; but this situation is unstable and, provided certain conditions are satisfied, will resolve into a stable pepper-and-salt pattern where some cells express Delta and Atoh1strongly ( the so-called primary fate) while other cells – their neighbours – experience inhibition and do not express Delta or Atoh1 (the secondary fate). We can describe the dynamics of this feedback control system and the emergence of the pattern by a set of delay differential equations [47], specifying how levels of NICD, *Delta* mRNA, *Hes* mRNA, and *Atoh1* mRNA in each cell change with time as a function of the levels of those same molecules in each cell of the system; full details of the computation are given as Text S2. We represent the effect of *Dll1* knockout as a reduction in the effective number of copies of *Delta* genes in each cell (assuming functional redundancy among the members of the *Delta* gene family). For large values of this parameter (large *Delta* gene dosage), the system (with hexagonal packing of the cells) robustly generates a standard 1∶3 pepper-and-salt mixture of primary- and secondary-fate cells (Figure 7B,C). At very low or zero *Delta* gene dosage, lateral inhibition fails and all cells uniformly adopt the primary fate (Figure 7G). Between these two extremes, we find a region of parameter space in which reduction of the *Delta* gene dosage leads to a graded increase in the proportion of cells that adopt the primary fate - that is, show high expression of *Atoh1* (Figure 7D–F). In other words, the model fits the observations.

### Diversification of secretory fate follows within much less than a cell cycle time after onset of Dll1 expression

This still leaves us with a puzzle. If Notch signaling controls creation of the difference between secretory and absorptive cells, when and how do secretory cells become diversified into goblet, enteroendocrine, and Paneth cell types? One idea [11] is that Notch signaling might act repeatedly in successive cell generations to refine the fate specification, as in the development of *Drosophila* mechanosensory bristles [48]. But this would not fit our finding that gut cells stop dividing as soon as they become committed to a secretory fate.

Indeed, our quantitative analysis of the S-phase labeling index, based on calculations taking account of the details of population dynamics and cell cycle behaviour in the crypt (Text S1), indicates that the choice between alternative secretory fates, as marked by Neurog3 and Gfi1, is decided within five hours or less after onset of Dll1 expression – a time much shorter than a cell cycle. The whole package of events – expression of Dll1, cessation of cycling, commitment to be secretory, and choice of specialised secretory fate – all go hand-in-hand, or at least follow hard upon one another's heels.

### Different levels of Delta-Notch signaling may correspond to different choices of secretory sub-type

What then does drive the diversification of secretory cell types? In the *Drosophila* intestine, where each cell faces a three-way choice between stem-cell, enterocyte, and enteroendocrine fates, these appear to correspond to different levels of Notch activation [49]. Could it be that in the mammalian gut too, the different cell types correspond not to a sequence of binary choices in successive cell generations, but to a single multi-way choice governed by Notch signaling?

Our mathematical model (Text S2) shows that this could indeed be the case. At appropriate *Delta* gene dosages, the lateral-inhibition circuitry can result in patterns where cells persistently expressing *Atoh1* and *Delta* touch one another, as well as occurring in isolation (Figure 7D–F). When such cells do touch, and only then, they will express *Hes* in addition to *Atoh1* and *Delta*. This results in a lower level of Atoh1 than in the isolated Atoh1 cells. According to this graded-Atoh1 model, isolated cells that express high levels of Atoh1 (and no Hes) would become goblet cells, cells expressing no Atoh1 (and high Hes) would become absorptive cells, and cells expressing an intermediate level of Atoh1 (and some Hes) would differentiate as enteroendocrine cells. (For Paneth cells, the evidence points to a different mechanism: their differentiation is apparently driven by the combination of a low or zero level of Notch activation, leading to Atoh1 expression, combined with exposure to very high levels of Wnt signaling [50].)

The graded-Atoh1 model would explain the observation [37] that enteroendocrine cells often arise in pairs, as well as our own observation that, when *Dll1* is knocked out, the frequency of enteroendocrine (Chga-positive) cells increases by an even larger factor than the frequency of goblet cells - a result that might otherwise seem surprising, given that all cells become goblet cells when Notch signaling is blocked completely. Further suggestive evidence comes from experiments where Atoh1 (Math1) was artificially expressed in the intestinal epithelium of mouse fetuses developing from eggs injected with a *villin:Atoh1* transgene [41]. The phenotype of the mice was variable, presumably because the tissues were mosaic with regard to presence of the transgene and variable in level of expression because of position effects due to different sites of chromosomal integration. In some individuals, virtually every cell in the intestinal epithelium became a goblet cell. In others, enteroendocrine cells (marked by Neurog3 expression) were increased more than 100-fold. This type of variation is consistent with the idea that the fate of the cells depends on the level at which the Atoh1 transgene is expressed.

Of course, our simple mathematical model of Notch signaling in the gut epithelium leaves many things out of account, including the role of Wnt signaling in maintaining expression of Notch pathway components, the occurrence of cell division within the population of interacting cells, and the pattern of cell migration out of the crypt. Other factors, such as the level of Wnt pathway activation at the time of commitment, may also contribute to the diversity of secretory fates and almost certainly do so in the case of Paneth cells [50]. The graded-Atoh1 hypothesis for secretory cell diversification thus remains speculative, and further experiments will be needed to test it. But whatever the outcome of such experiments may be, the major experimental finding of the present paper - that expression of Delta, secretory commitment, secretory specialisation, and exit from the cell cycle all go hand-in-hand in the intestinal stem-cell system - takes us a significant step closer to a detailed understanding of how the diverse cell types in the lining of the gut are generated in the observed proportions.

## Methods

### Ethics statement

Animal experiments were approved by the CRUK London Research Institute Ethical Review Committee (ref JLE1706) and performed in conformity with UK Home Office Project License 80/2081 held by JL.

### Mouse lines

*Dll1^+/lacZ^* mice, carrying an insertion of *lacZ* into the *Dll1* locus, were as described in [18], and bred onto a C57Bl6/J genetic background. For conditional knockout of *Dll1*, we used mice carrying a floxed allele of *Dll1* (*Dll1^flox^*) as described in [25], [26]. These mice were bred to carry also the *AhCre* transgene, giving Cre expression in the gut in response to β-naphthoflavone [27]. Adult (3 to 6 months old) *Dll1^flox/flox^*;*AhCre* mice and control littermates lacking the *AhCre* transgene received intraperitoneal injections of β-naphthoflavone (10 µl per g body weight of 8 mg/ml solution in corn oil) on three consecutive days and were killed for analysis 5, 12 or 28 days after the first injection.

### Proliferation assay

EdU (5-ethynyl-2′-deoxyuridine) was injected intraperitoneally (10 mg/ml in PBS, 10 µl per g of body weight) one hour before animals were killed. EdU was detected using the Click-iT assay (Invitrogen C10339) according to manufacturer's instructions. Where EdU detection was combined with immunostaining of frozen sections (Figure 5C), the EdU detection step was performed first. Where paraffin sections were used (Figure 5D–E), antigen retrieval and immunostaining was done first, followed by the EdU detection step.

### Wax histology and immunohistochemistry

For wax histology, the intestine was dissected, formalin-fixed, embedded and sectioned as in [51]. Sections were immunostained using the primary antibodies anti-Mucin-2 (Santa Cruz sc15334; 1∶200) and anti-chromogranin (Abcam ab151601; 1∶1250), all diluted in 1% BSA/PBS, following antigen retrieval in citrate buffer for 15 minutes. Secondary antibody was biotinylated goat anti-rabbit, detected with the ABC system (Vector Laboratories PK-6100) and DAB chemistry to give a brown stain. Slides were counterstained with haematoxylin. To show mucus in goblet cells, tissue sections were stained with alcian blue and counterstained with Mayer's haematoxylin.

### Cryosectioning

For frozen sectioning, the whole length of the small intestine was divided into three segments, flushed with cold PBS, and fixed in 4% paraformaldehyde in PBS for 3–4 hours at 4°C. After a rinse in PBS the tissue was cryoprotected overnight at 4°C in 30% sucrose in PBS, embedded in OCT, and sectioned at 15 µm.

### Immunofluorescence

For immunofluorescent staining of cryosections, we used a blocking solution of 1% BSA and 0.1% Triton X-100 in PBS and the following primary antibodies and lectins: chicken anti-β-galactosidase (Abcam ab9361, 1∶1000), goat anti-Dll4 (R&D Systems AF1389, 1∶100), goat anti-Jag1 (Santa Cruz C-20, 1∶100), goat anti-Jag2 (Santa Cruz R-19, 1∶100), wheat-germ-agglutinin-Alexa488 (Molecular Probes W11261, 1∶100), rabbit anti-chromogranin A (Diasorin 20085, 1∶1000), rabbit anti-lysozyme (1∶100, Novocastra). Wax sections were stained with: rabbit anti-β-catenin (Sigma C2206, 1∶1250), rabbit anti-β-galactosidase (2BScientific R1064P, 1∶4000), goat anti-Gfi1 (Santa Cruz Biotechnology sc-8558, 1∶75), rabbit anti-NICD (Abcam ab8925, 1∶250), and mouse anti-Neurog3 (Developmental Studies Hybridoma Bank, University of Iowa, f25a1b3, 1∶40). Secondary antibodies were: Alexa-conjugated anti-goat, anti-mouse and anti-rabbit (Invitrogen), and FITC-conjugated anti-chick (Jackson Immunoresearch). Nuclei were stained with 4′-6-diamidino-2-phenylindole (DAPI), or DRAQ5 (Biostatus Ltd). Frozen sections were mounted in Slowfade (Invitrogen); wax sections were mounted in HardSet (Vector Laboratories). Images were captured using Zeiss LSM510, LSM700 and LSM710 confocal microscopes.

### *In situ* hybridisation

Tissues were fixed in 4% neutral buffered formalin for a maximum of 24 hours, embedded in paraffin wax, and sectioned at 5 µm. *Hes1* mRNA was detected by in situ hybridisation using ^35^S-UTP labeled antisense riboprobes and autoradiographs were prepared essentially as in [52]. Results were photographed on a Nikon ME600 microscope with bright field (to show the stained tissue) and with dark-field epi-illumination (to show silver grains), and the dark-field image was superimposed in false color on the bright field image using Adobe Photoshop.

### RNA preparation and qRT-PCR

Small segments (about 1 cm) of anterior jejunum were isolated from the rest of the small intestine, opened longitudinally, rinsed in cold PBS and then incubated in 30 mM EDTA in PBS (Mg^2+^ and Ca^2+^ free) at 4°C for two hours. The epithelium was isolated from the underlying tissue by gentle stroking using forceps, then rinsed with cold PBS to remove the EDTA, and frozen on dry ice.

Total RNA was prepared from the isolated intestinal epithelium using the SV Total RNA Isolation System (Promega). Reverse transcription (RT) was performed at 42°C for one hour using the Retroscript kit (Ambion). The resulting cDNA was analysed by real-time PCR using Platinum SYBR Green qPCR Supermix (Invitrogen) on a Chromo4 apparatus (BioRad) with Opticon Monitor 2 software. For each PCR, 0.1 µl of the relevant cDNA preparation was used as template. The efficiency of each primer pair was checked using a cDNA dilution series. Each reaction was performed in duplicate. mRNA levels relative to β*-actin* were calculated from Ct values according to the 2^(Ctβ*actin*-Ct*target*)^ formula. Each data point is the mean of 5 or 6 mice. Error bars show standard error of the mean.

Primers for qRT-PCR were:

β-actin F: 5′-GGAAGGTGACAGCATTGCTTC-3′

β-actin R: 5′-GGTCTCAAGTCAGTGTACAGG-3′

Dll1 F: 5′-CCCATCCGATTCCCCTTCG-3′

Dll1 R: 5′-GGTTTTCTGTTGCGAGGTCATC-3′

Dll4 F: 5′-CAGTTGCCCTTCAATTTCACCT-3′

Dll4 R: 5′- AGCCTTGGATGATGATTTGGC-3′

Jag1 F: 5′-AAAGTGTGCCTCAAGGAGTATCA-3′

Jag1 R: 5′-TGAGATTGAAGGTGTTACCCCC-3′

Hes1 F: CAGCTGATATAATGGAGAAAAATTCCT

Hes1 R: TTGTCCGGTGTCGTGTTGAC

Hes5 F: 5′-CTGGAGATGGCCGTCAGCTACCTG-3′

Hes5 R: 5′-GAGTAGCCCTCGCTGTAGTCCTG-3′

Atoh1 F: 5′-GCTGGACGCTTTGCACTTC-3′

Atoh1 R: 5′-TCTGTGCCATCATCGCTGTT-3′

### Statistics

We used a one-tailed Student's t-test to calculate statistical significance of differences between cases.

### Mathematical modeling

We used *Mathematica* for our calculations of labeling index (Text S1) and to model the behavior of a hexagonal array of cells interacting through Delta-Notch signaling (Text S2). The latter model represents the state of each cell at any instant by its concentrations of *Hes* mRNA, *Atoh* mRNA, *Delta* mRNA, and NICD protein, with dynamics described by delay differential equations and parameter values loosely based on data from other systems [47], [53], [54]. The qualitative conclusions are relatively insensitive to the parameter choices. Calculations shown in Figure 7 assume a 10×10 hexagonally packed sheet of cells, with cyclic boundary conditions and an initial state in which all the genes are expressed but at random low levels. For full details, see the program itself, provided in PDF format as Text S2. The executable Mathematica notebooks are available by email from julian.lewis@cancer.org.uk.

## Supporting Information

Text S1 **S-phase labelling index and its relation to a Dll1-associated cell-cycle block: a calculation for the mouse small intestine.** We are interested in the cell-cycle behaviour of those cells that have switched on expression of the Notch ligand Dll1, as manifest in expression of our β-galactosidase reporter, and are present in the intestinal crypt at the time of analysis. We have measured the fraction of these cells that are found in S-phase of the cell division cycle, by labelling for a short period (1 hour) with EdU and fixing immediately afterwards. To interpret our measurements, we want to calculate the expected S-phase labelling index for this cell population, that is, the fraction *f* that are expected to be labelled with EdU following a short pulse of exposure, on the assumption that expression of Dll1 is associated with a block to cell cycling. In making this calculation, we have to allow for the fact that any cell that has passed a certain point in the cell cycle, called Start, is committed to go on through S phase and complete a division cycle and will do so even if acted upon by a stop signal associated with onset of Dll1 expression. The calculation is done with Mathematica, using published data for the cell-cycle parameters of intestinal crypt cells.
(PDF)

Text S2 **A model of Delta-Notch-mediated lateral inhibition in a sheet of intestinal epithelial cells: Effects of varying** ***Delta*** **gene dosage.** This Mathematica program computes the behavior of an array of cells interacting with one another via the Delta-Notch lateral-inhibition pathway as indicated by the gene-regulatory circuit diagram shown in Figure 7. The cells are assumed to start off all in a similar state, but with some minor random variation from one cell to the next. The computation shows that the pattern of cell states that ultimately emerges depends on the number of functional *Delta* gene copies that the cells contain, in the manner summarised in Figure 7.
(PDF)
