# Supplementary material for: Delta1 Expression, Cell Cycle Exit, and Commitment to a Specific Secretory Fate Coincide within a Few Hours in the Mouse Intestinal Stem Cell System
Source: PLoS One. 2011 Sep 7;6(9):e24484. doi: 10.1371/journal.pone.0024484 (PMC3168508; doi:10.1371/journal.pone.0024484)

# Supporting Information S1

## S-phase labelling index and its relation to a Dll1-associated cell-cycle block: a calculation for the mouse small intestine

We are interested in the cell-cycle behaviour of those cells that have switched on expression of the Notch ligand Dll1, as manifest in expression of our $\beta$-galactosidase reporter, and are present in the intestinal crypt at the time of analysis. We have measured the fraction of these cells that are found in S-phase of the cell division cycle, by labelling for a short period (1 hour) with EdU and fixing immediately afterwards. To interpret our measurements, we want to calculate the expected S-phase labelling index for this cell population, that is, the fraction $f$ that are expected to be labelled with EdU following a short pulse of exposure, on the assumption that expression of Dll1 is associated with a block to cell cycling. In making this calculation, we have to allow for the fact that any cell that has passed a certain point in the cell cycle, called Start, is committed to go on through S phase and complete a division cycle and will do so even if acted upon by a stop signal associated with onset of Dll1 expression.

Let a typical secretory-lineage cell switch on Dll1 (i.e. acquire the visible $\beta$-galactosidase label) at time $d$.
Define $t = 0$ to be the time of EdU pulse-labelling and fixation (assumed to be a single point in time).
Let the cell (or cell lineage, if the cell divides) exit from the crypt (i.e. from the population of interest) at time $d + r$ , where $r$ is the average crypt residence time for Dll1-expressing cells.
The population that interests us then consists of those cells for which
$0 > d > -r$.
Let $T$ be the cell cycle time.
Define the cell-cycle phase of a cell to be the time that has elapsed since the moment of cell division, i.e. since the beginning of G1 phase at cytokinesis. Phase will thus be a variable lying between 0 and T.
Let $u$ be the cell cycle phase corresponding to onset of DNA synthesis.
Let $v$ be the cell cycle phase corresponding to completion of DNA synthesis.
Let $q$ be the Start point in the cell cycle, such that a cell having passed this point is already committed to proceed into S phase and complete its current division cycle, regardless of any signal that may be imposed to halt cell cycling.

For generality, we suppose that onset of Dll1 expression at time $d$ creates a block to initiation of the cell division cycle, but that this block comes into force with a delay $b$, i.e. at a time $d + b$. The block then acts by prohibiting progress past Start. The block is unable to stop the cell from progressing into or through a final S phase if the cell has already passed Start. Note that $b$ does not have to be positive: negative values of $b$ describe the case where the block to cell cycling precedes the onset of Dll1 expression.

Let $x$ be the cell-cycle phase of the given cell at time $d + b$, i.e. at onset of the block.
If $x > q$, the final S phase will start at time $d + b + u - x$ and will end at $d + b + v - x$.
If $x \leq q$, no further S phase will ensue after onset of the block (since the block has been imposed before passage past Start) and the final S phase will be the preceding one, which began at time $d + b - T + u - x$ and ends at $d + b - T + v - x$.
Thus the cell (or cell lineage) will have a set of S phases occupying the time intervals

$$s(x, d) = \begin{cases} \{(d + b + u - x - nT,\ d + b + v - x - nT), \text{ for } n = 0, \ldots\infty\} \text{ if } x > q \\ \{(d + b + u - x - nT,\ d + b + v - x - nT), \text{ for } n = 1, \ldots\infty\} \text{ if } x \leq q \end{cases}$$

The cell/cell lineage will be in the crypt population of Dll1-expressing cells at the time of labelling/fixation (time 0) if and only if
$-r < d < 0$ Condition C1
and it will at the same time be labelled with EdU if and only if the time of labelling/fixation (time 0) lies within an S phase, i.e. if and only if

$0 \in s(x, d)$ Condition C2

The condition C2 means that $x$ must be such that

$x > q$ and for some $n \in [0, \infty)$, $d + b + u - x - \text{nT} < 0 < d + b + v - x - n\,T$
OR
$x \leq q$ and for some $n \in [1, \infty)$, $d + b + u - x - \text{nT} < 0 < d + b + v - x - n\,T$

This boils down to the condition:

$x > q$ and $d + b + u < x < d + b + v$
OR
for some $n \geq 1$, $d + b + u - n\,T < x < d + b + v - n\,T$ Condition C2'

There is an upper limit to the range of possible values of $n$. To see this, note that to satisfy the second half of the above condition (following the OR) we must have
$0 < d + b + v - x - n\,T$.
Since $d < 0$ and $x > 0$, this implies
$n < (b + v)/T$.
Thus the values of n that could satisfy the second half of Condition C2 must lie within the range
$1 \leq n \leq n_{\max}$
where
$n_{\max}$ is the greatest integer less than $(b + v)/T$.

Condition C2 can therefore be restated as
$x > q$ and $d + b + u < x < d + b + v$
OR
for some $n \in [1, n_{\max}]$, $d + b + u - n\,T < x < d + b + v - n\,T$

Let $p(x, d)$ be the probability density for a Dll1-expressing cell (or its ancestor) to have switched on Dll1 at time $d$ and to have phase $x$ at the time of onset of the block. Then the fraction $f(r, b, q)$ of Dll1-expressing cells in the crypt that are EdU-labelled (i.e. the labelling index for these cells), for any given values of $r$, $b$ and $q$, will be the integral over
all possible values of $d$ and $x$ that satisfy Conditions C1 and C2, weighted by the probability density $p(x, d)$ for a cell to be in that region of $(x, d)$ space:

$$f(r, b, q) = \int_{-r}^{0} \int_{0,\, x \text{ satisfying Condition C2}}^{T} p(x, d)\, dx\, dd$$

To work out the form of $p(x, d)$, we have to take account of two factors.

First, if a Dll1-expressing cell divides to give two cells, this doubles the representation of that cell lineage in the population. We can assume that cell lineages expressing Dll1 are initiated at a steady rate. It then follows that the number of Dll1-expressing cells in a crypt that have $d$ in the interval $(d,\ d + \delta\, d)$, for any given $x$, will be proportional to $\delta\, d * n_c(x, d)$ where $n_c(x, d)$ is the number of cells at the time of fixation in the clone generated by cell divisions from an $(x, d)$ founder cell. In other words, for any given $x$, $p(x, d)$ must be proportional to $n_c(x, d)$.

Second, $p(x, d)$ has to take account of the fact that cells are non-uniformly distributed over $x$. To see why this must be so, note that we must have $p(0, d) = 2\, p(T, d)$, since the number of cells just emerging from cytokinesis must always be twice the number just entering. In fact, if we assume that the proliferative cells in the crypt have a steady-state distribution over the phases of the cell cycle, a bit of standard theory shows that we must have $p(x, d) = 2^{-x/T}\, p(0, d)$ (see, for example, Wright and Allison, 1984).

Combining these factors, we see that the joint probability density $p(x, d)$ is proportional to $n_c(x, d)\, 2^{-x/T}$.

Defining the normalisation factor

$A = \int_{-r}^{0}\int_{0}^{T} n_c(x, d)\, 2^{-x/T}\, dx\, dd$

we thus have

$p(x, d) = (1/A)\, n_c(x, d)\, 2^{-x/T}$

To evaluate $n_c(x, d)$, we consider separately the cases $x < q$, where the block acts before Start, and $x \geq q$, where it acts after.

**For $x > q$**, the last division will occur at $d + b + T - x$ and this will be preceded by earlier divisions of the Dll1-expressing lineage at times $d + b + T - x - i\,T$ ( for $i = 1, 2, 3, \ldots$) . In other words, the cell lineage will undergo divisions at times $t_{\text{div}}(j) = d + b - x - j\,T$ ( for $j = -1, 0, 1, 2, \ldots$). Some of these divisions, however, will be irrelevant to our calculations because they occur before the onset of Dll1 expression or after the time of fixation. The relevant divisions - those that occur after the onset of Dll1 expression and before the time of fixation - will be the ones for which

$j \geq -1$ and $d < t_{\text{div}}(j) < 0$,

i.e.

$j \geq -1$ and $d < d + b - x - j\,T < 0$

i.e.

$j \geq -1$ and $d + b - x < j\,T < b - x$

i.e.

$-1 \leq j$ and $j_{\min} \leq j \leq j_{\max}$

where $j_{\min}$ is the smallest integer greater than $(d + b - x)/T$ and $j_{\max}$ is the greatest integer smaller than $(b - x)/T$. Defining

$j_{\text{maxmin1}} = \text{Max}(-1, j_{\min})$

we see that, for $x > q$, the total number of divisions undergone by an $(x, d)$ lineage in the crypt from onset of Dll1 expression up to the time of fixation is

$$n_{\text{div1}}(x, d) = \begin{cases} j_{\max} - j_{\text{maxmin1}} + 1 & \text{for } j_{\max} \geq j_{\text{maxmin1}} \\ 0 & \text{for } j_{\max} < j_{\text{maxmin1}} \end{cases}$$

**For $x \leq q$**, the last division will occur at $d + b - x$ and this will be preceded by earlier divisions of the Dll1-expressing lineage at times $d + b - x - i\,T$ ( for $i = 1, 2, 3, \ldots$). By an argument similar to that used above, we find that in this case, for $x \leq q$, the total number of divisions undergone by an $(x, d)$ lineage in the crypt from onset of Dll1 expression up to the time of fixation is

$$n_{\text{div2}}(x, d) = \begin{cases} j_{\max} - j_{\text{maxmin0}} + 1 & \text{for } j_{\max} \geq j_{\text{maxmin0}} \\ 0 & \text{for } j_{\max} < j_{\text{maxmin0}} \end{cases}$$

where

$j_{\text{maxmin0}} = \text{Max}(0, j_{\min})$

Combining these results, we conclude that the number of cells in an $(x, d)$ lineage at the time of fixation is

$$n_c(x, d) = \begin{cases} 2^{n_{\text{div1}}(x,d)} & \text{if } x > q \\ 2^{n_{\text{div2}}(x,d)} & \text{if } x \leq q \end{cases}$$

Data on cell cycle parameters for mouse small intestine are summarised in N. Wright and M. Allison, “The Biology of Epithelial Cell Populations” Vol 2, Table 18.3, page 650 (Oxford: Clarendon Press, 1984). There is some variation between results from different studies, but consensus values are approximately as follows:

$T$ = cell cycle time = 13 hours

$u$ = G1 phase = 4 hours
$v - u$ = S phase = 7 hours
G2 phase = 1.2 hours
M phase = 0.8 hours.
The crypt residence time $r$ for Dll1-expressing cells is less well defined but should be less than or of the order of 25 hours (see main text).

The following *Mathematica* program implements the above calculations, with the above data, to compute the predicted labelling index, i.e. the fraction $f(r, b, q)$ of Dll1-expressing cells in the crypt that are EdU labelled, for any given values of $r$, $b$ and $q$.

```
hour = 1.;
T = 13 hour;
u = 4 hour;
v = u + 7 hour;
condition2[xc_, dc_, bc_, qc_] :=
  (
   nmax = Floor[(bc + v) / T];
   Or[(xc > qc && (dc + bc + u < xc < dc + bc + v)),
    Apply[Or, Table[dc + bc + u - xc - n T < 0 < dc + bc + v - xc - n T, {n, 1, nmax}]]]
  );
f[r_, b_, q_] :=
 (
  nc1[xn1_, dn1_] :=
   (
    jmax = Floor[(b - xn1) / T];
    jmaxmin1 = Max[-1, Ceiling[(b - xn1 + dn1) / T]];
    2^Sum[1, {j, jmaxmin1, jmax}]
   );
  nc2[xn2_, dn2_] :=
   (
    jmax = Floor[(b - xn2) / T];
    jmaxmin0 = Max[0, Ceiling[(b - xn2 + dn2) / T]];
    2^Sum[1, {j, jmaxmin0, jmax}]
   );
  nc[xn_, dn_] := If[xn > q, nc1[xn, dn], nc2[xn, dn]];
  pp[xp_, dp_] :=
   nc[xp, dp] 2^(-xp/T);
  NIntegrate[If[condition2[x, d, b, q], 1, 0] pp[x, d], {x, 0, T}, {d, -r, 0}] /
   NIntegrate[pp[xpp, dpp], {xpp, 0, T}, {dpp, -r, 0}]
 )
```

Note that the labelling index in the limit where residence time $r \to 0$, with $b \geq 0$, is equal to that for the non-Dll1-expressing cell population, since $r \to 0$ describes the case where the Dll1-expressing cells are labelled and fixed immediately at the time of onset of Dll1 expression, and at this moment, provided $b \geq 0$, they have the same chance as any other cell to be in S-phase. Thus the labelling index for the population of crypt cells that do not express Dll1 is predicted to be

$f_0 = \lim_{r \to 0} f(r, 0, q)$,

which evaluates, using the above program, to

$f_0 = 0.503$

This is somewhat larger than our experimental value, which was 0.38±0.03. The discrepancy has a simple explanation. Our theoretical calculation assumes that the whole crypt is a uniformly proliferative compartment, where the only block to cell division is that associated with onset of Dll1 expression. In fact, the uppermost part of the crypt, corresponding to the topmost ~6 cell positions (out of 25) is a maturation zone where few or no S-phases are seen even among the future absorptive cells (Wright and Allison, p 649-651). Here, presumably, the environment is no longer permissive for cell division. The foregoing theoretical calculations should be correct for the proliferative zone below this, but our experimental counts of the S-phase labelling index represent averages over the whole crypt, including the non-

proliferative maturation zone, whose inclusion dilutes the proportion of S-phase cells. The theoretical predictions (which are based on cell-cycle parameters for the proliferative zone) should therefore be scaled down by a correction factor of roughly 19/25, i.e. roughly 0.76, for comparison with our measurements; and this correction factor should apply equally to the predictions for both classes of cells - those that express Dll1 and those that do not. The raw prediction for the labelling index for cells not expressing Dll1 is 0.503; our experimental value was 0.38 ± 3%, implying that the precise value of the appropriate correction factor is in fact 0.76 ± 0.06. Applying this same factor to our calculations for the labelling index of the Dll1-expressing population, our corrected prediction for the EdU-labelling index of the population of Dll1-expressing cells in the crypt is

```
fcor[r_, b_, q_] := 0.76 f[r, b, q]
```

giving the predicted values shown in the graphs below:

```
gg = Table[Plot[
    {fcor[r, b, q], 0.07}, {b, -6, 6},
    PlotRange → {0, 0.5},
    PlotPoints → 50,
    MaxRecursion → 0,
    Frame → True,
    FrameLabel →
     {Style["delay b (hours)", FontFamily → "Arial", FontSize → 16],
      Style["labelling index f", FontFamily → "Arial", FontSize → 16]},
    AspectRatio → 1,
    FrameTicks → True,
    PlotLabel → Style[StringJoin["\nr=", ToString[r], "   q=", ToString[q]],
      FontFamily → "Arial", FontSize → 16]
   ], {r, 15, 25, 5}, {q, 0, 4, 2}];
Print[TableForm[gg]]
```

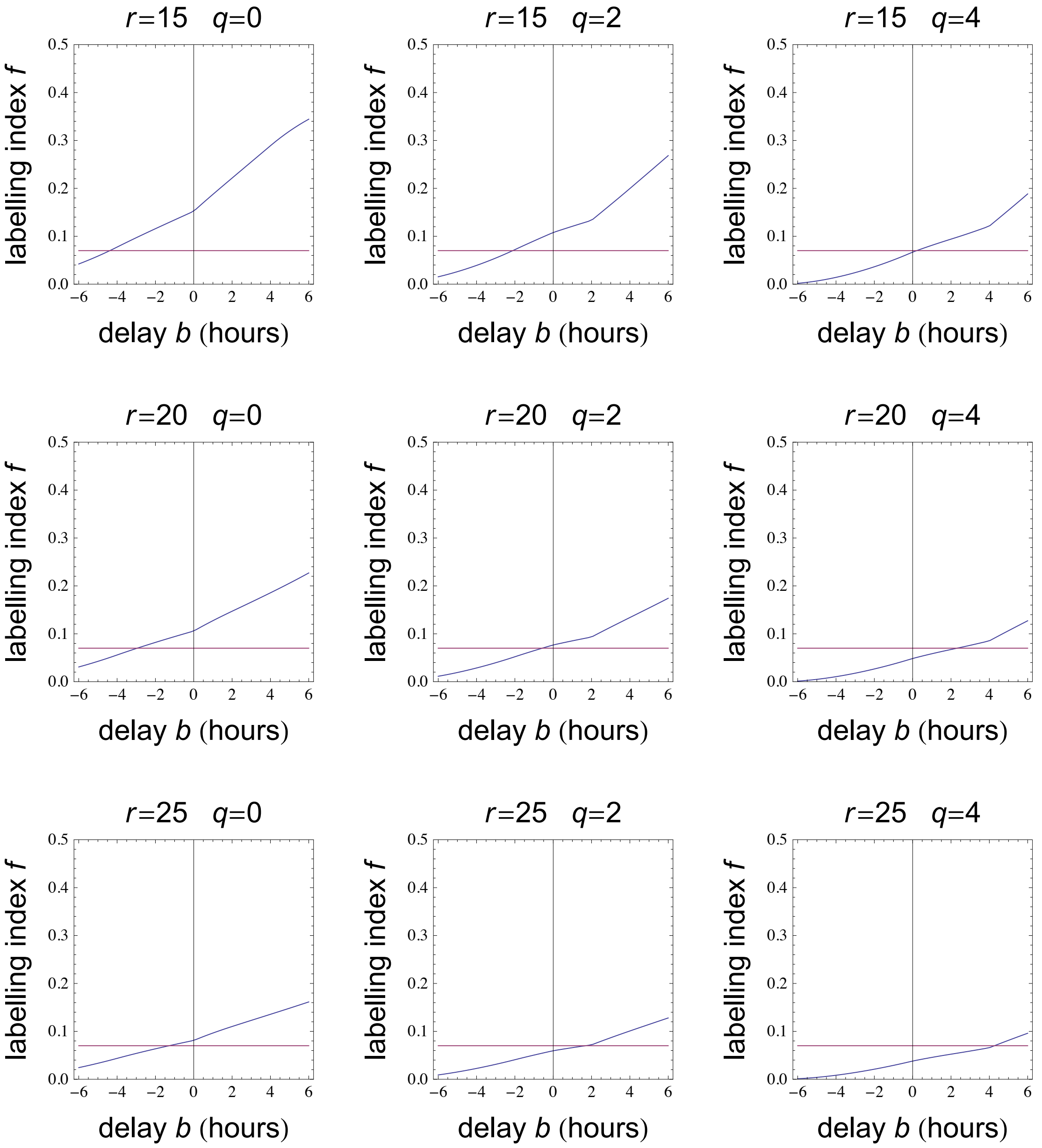


In these graphs, the horizontal line represents our experimental value for the EdU pulse-labelling index of crypt cells expressing the Dll1 reporter, while the curve shows their predicted labelling index *f*, as a function of the delay *b* from onset of reporter expression to onset of the block to cell cycling. Results are shown for each of a range of possible values of the cells' average crypt residence time *r* (taken to be

somewhere between 15 and 25 hours) and of the timing $q$ of Start in the cell cycle (taken to lie somewhere between the beginning of G1 phase ($q = 0$) and the beginning of S phase ($q = 4$ hours)). From the point of intersection of the line and the curve, we can read off the value of the delay that must be postulated to explain the observed labeling index on the assumption of the specified values of $r$ and $q$. No matter what values we assume for $r$ and $q$ within these plausible ranges, we see that the delay $b$ cannot be more than ±4 hours.

Finally, we wish to interpret the difference in measured labelling index between cells expressing the Dll1 reporter (labelling index 0.07) and those expressing Neurog3 (labelling index 0.036) or Gfi1 (labelling index 0.014). The same arguments used above to relate the labelling index to the timing of Dll1 expression in relation to a block to cell cycling can be equally well applied to the timing of any other marker in relation to the same block. The lower labelling indices for Neurog3 and Gfi1 imply that these markers switch on with a delay relative to Dll1, so that by the time of their appearance fewer cells will be caught in S phase, because the block has been in force for a longer time. The graph below enables us to make quantitative statements about these relative delays. In the graph, we plot the dependence of labelling index on the delay $b$, for representative values of $q$ (set at 2 hours) and $r$ (set at 20 hours). By comparing with the measured values of the labelling index for the different markers, we can deduce approximate values for their relative times of onset. The horizontal lines represent the measured values of the labelling index for each of the three markers, and the inferred delays are read off from the points of intersection of these lines with the theoretical labelling index curve.

```
Plot[{fcor[20, b, 2], 0.07, .036, .014}, {b, -8, 8},
 PlotRange → {0, 0.1}, PlotPoints → 50,
 MaxRecursion → 0, Frame → True, FrameLabel →
  {Style["delay b (hours)", FontFamily → "Arial", FontSize → 16], Style[
    "labelling index f", FontFamily → "Arial", FontSize → 16]}, AspectRatio → 1]
```

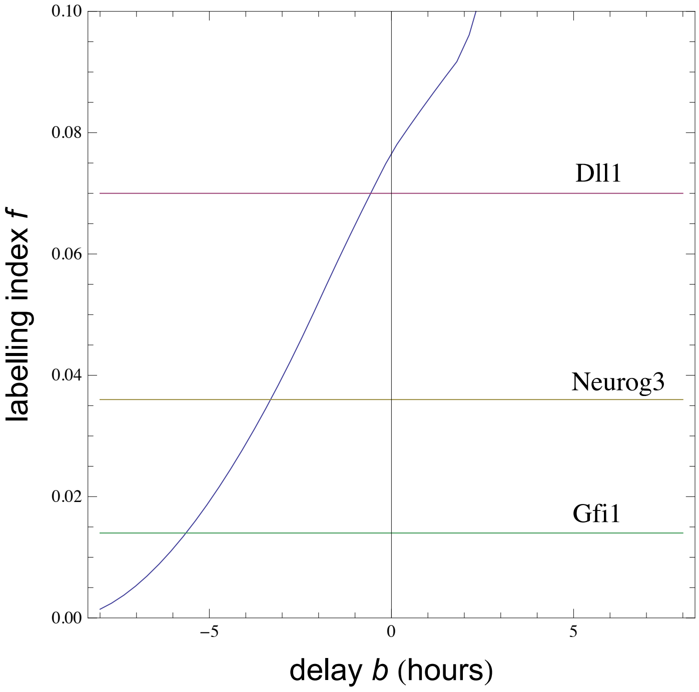

Supplement: Text S1 — S-phase labelling index and its relation to a Dll1-associated cell-cycle block: a calculation for the mouse small intestine. We are interested in the cell-cycle behaviour of those cells that have switched on expression of the Notch ligand Dll1, as manifest in expression of our β-galactosidase reporter, and are present in the intestinal crypt at the time of analysis. We have measured the fraction of these cells that are found in S-phase of the cell division cycle, by labelling for a short period (1 hour) with EdU and fixing immediately afterwards. To interpret our measurements, we want to calculate the expected S-phase labelling index for this cell population, that is, the fraction f that are expected to be labelled with EdU following a short pulse of exposure, on the assumption that expression of Dll1 is associated with a block to cell cycling. In making this calculation, we have to allow for the fact that any cell that has passed a certain point in the cell cycle, called Start, is committed to go on through S phase and complete a division cycle and will do so even if acted upon by a stop signal associated with onset of Dll1 expression. The calculation is done with Mathematica, using published data for the cell-cycle parameters of intestinal crypt cells. (PDF) [file pone.0024484.s001.pdf]
