# Supplementary material for: Delta1 Expression, Cell Cycle Exit, and Commitment to a Specific Secretory Fate Coincide within a Few Hours in the Mouse Intestinal Stem Cell System
Source: PLoS One. 2011 Sep 7;6(9):e24484. doi: 10.1371/journal.pone.0024484 (PMC3168508; doi:10.1371/journal.pone.0024484)

Supporting Information S2

A model of Delta-Notch-mediated lateral inhibition in a sheet of intestinal epithelial cells: Effects of varying *Delta* gene dosage.

To keep the file size manageable, we here show the program evaluated for an array of only 20 cells, and for just 3 values of the Delta gene dosage.

- **List the molecules involved and give each one an index number**

```
moltypes = {"matoh", "mdelta", "mhes", "pnicd"};
nmols = Length[moltypes];
imatoh = 1;
imdelta = 2;
imhes = 3;
ipnicd = 4;
```

- **Set the timespan of simulation and the number of elementary time-steps corresponding to one minute**

```
timestep = 1;
minute = 1 * timestep;
tfinal = Round[4000 minute];
```

- **Define the size of the system (n1 cells x n2 cells).**

```
n1 = 5;
n2 = 4;
```

- **Define the geometry of the system and assign an index number to each cell. Specify the topology by listing the neighbours of each cell.**

```
latticevector1 = N[{Sqrt[3], 0}];(* for hexagonal lattice *)
latticevector2 = N[{Sqrt[3] / 2, 3 / 2}]; (* for hexagonal lattice *)

addresses = Flatten[Table[{ja, jb}, {ja, 0, n1 - 1}, {jb, 0, n2 - 1}], 1];
(* list of lattice addresses of the cells in the patch *)

ncells = Length[addresses];
index[{ja_, jb_}] := jb + 1 + n2 * ja;
(*  serial number of cell at lattice address {ja,jb} *)

neighbouraddresses[jcell_] :=
 ({j1, j2} = addresses[[jcell]];
  {{Mod[j1 + 1, n1], j2}, {Mod[j1 - 1, n1], j2}, {j1, Mod[j2 + 1, n2]}, {j1, Mod[j2 - 1, n2]},
   {Mod[j1 - 1, n1], Mod[j2 + 1, n2]}, {Mod[j1 + 1, n1], Mod[j2 - 1, n2]}})
(* hexagonal lattice, with cyclic boundary conditions *)

Table[neighbourindices[jcell] = Flatten[index /@ neighbouraddresses[jcell]],
  {jcell, 1, ncells}];

xyposition[jcell_] :=
  latticevector1 * addresses[[jcell, 1]] + latticevector2 * addresses[[jcell, 2]] ;
```

- **Specify the delays and molecular lifetimes.**

The next program segment specifies the delays as a set of values *delay[[target, agent]]*, meaning that the rate of change of the "target" molecule at time *t* is determined by the value of the "agent" molecule at time *t - delay[[target,agent]]*. In other words, *delay[[target,agent]]* is the delay from making a change in the quantity of *agent* to obtaining a resultant change in the quantity of *target*.
Delays may be different for actions in cis (same cell) and in trans (from neighbouring cells).
The program is set up to allow for different values of the delays and other parameters in different cells and at different times.

Irrelevance of delays in steady state:

Note that, although the delays are critical for the dynamical behaviour of the system, they do not enter into the equations that define the possible steady states (though they may determine whether the steady states are stable). With the parameter choices used below, our system does tend to a stable steady state, and it is this steady state that we take to represent the final pattern of cell fate choices.

Estimation of delays:
In general, if we are assuming that the protein product of gene A directly regulates the transcription of gene B, but we are representing this in terms of regulation of mRNA by mRNA, we must set the delay for mA to regulate mB to include the delay involved in production of the protein pA from the message mA.
We therefore estimate as follows:
delay[[mB, mA]]
= delay for production of pA from mA + transcriptional delay of mB
≈ translational delay for A + lifetime of pA + transcriptional delay of mB .
In this formula, the lifetime of pA can be thought of as an accumulation delay, to be added to the delay involved in making each individual protein molecule and delivering it to its site of action.
Thus:
• for matoh to regulate mdelta, the cis delay is
translational delay for atoh + lifetime of patoh + transcriptional delay for mdelta
• for matoh to regulate matoh, the cis delay is
translational delay for atoh + lifetime of patoh + transcriptional delay for matoh
• for mhes to regulate matoh, the cis delay is
translational delay for hes + lifetime of phes + transcriptional delay for matoh
• for mhes to regulate mdelta, the cis delay is
translational delay for hes + lifetime of phes + transcriptional delay for mdelta.
• For pnicd to regulate mhes, the cis delay is simply
transcriptional delay for mhes.
• For mdelta to regulate pnicd, the trans delay is
translational delay for delta (including time for delivery to cell surface) + lifetime of pdelta.

Notation:
In the assignments below,
tm.... denotes mRNA lifetime
tp... denotes protein lifetime (i.e. lifetime at its site of action)
dm... denotes transcriptional delay
dp... denotes translational delay (including time for maturation and delivery of protein to its site of action)

Values for delays and lifetimes below are loosely based on Lewis (Current Biol., 2003), Giudicelli et al. (PLoS Biol., 2007) and Hirata et al. (Genes Dev., 2004). A relatively long lifetime *tmatoh* for *atoh* mRNA is postulated, to prevent the system from oscillating.

```
tmatoh = 200 minute;
dmatoh = 10 minute;
tpatoh = 10 minute;
dpatoh = 2 minute;

tmhes = 10 minute;
dmhes =  30 minute;
tphes =  20 minute;
dphes =  2 minute;

tmdelta =  10 minute;
dmdelta =  10 minute;
tpdelta =  10 minute;
dpdelta =  30 minute;

tpnicd =  10 minute;
```

Specify the tables of cis and trans delays by first setting all to zero, and then specifying values for those that are non-zero.
First index of *cisdelay[[i,j]]* or *transdelay[[i,j]]* specifies target, and second index specifies regulatory molecule.

```
cisdelay = Table[0, {nmols}, {nmols}] minute;

cisdelay[[imatoh, imatoh]] = Round[tpatoh + dmatoh + dpatoh];
cisdelay[[imdelta, imatoh]] = Round[tpatoh + dpatoh + dmdelta];
cisdelay[[imatoh, imhes]] = Round[tphes + dphes + dmatoh];
cisdelay[[imdelta, imhes]] = Round[tphes + dphes + dmdelta];
cisdelay[[imhes, ipnicd]] = Round[dmhes];

transdelay = Table[0, {nmols}, {nmols}] minute;

transdelay[[ipnicd, imdelta]] = Round[dpdelta + tpdelta];

maxdelay = Max[Table[{cisdelay, transdelay}, {jcell, 1, ncells}, {t, 1, tfinal}]];
```

■ Specify rate constants, critical concentrations, and dynamical equations

For each kind of molecule, f0 specifies its concentration at the next time point as a function of the currently acting concentrations of the various types of molecules in the same cell (cisconcs) and in the neighbouring cells (transconcs). These "currently acting concentrations" are in general the values that were present at some earlier times, corresponding to delays in the control system, denoted by a prefix r (for retarded). However, sometimes - in particular when a molecule directly regulates its own synthesis, but with a delay - we may need to have f0 depend on both the current value of a concentration (cisconcs) and on its retarded value (rcisconcs). When f0 is called later in the program, it will be with the suitably delayed values of the concentrations as arguments. Note that the program allows for f0 to be different in different cells and at different times.

```
khes = 5. * 1/tmhes; (* khes*tmhes is the steady-state concentration of mhes,
when mhes is being made at its maximal rate. *)
katoh = 5. * 1/tmatoh;
kdelta = 0.2 * 1/tmdelta;
kn = 5. * 1/tpnicd;
katohExtra = .1 * katoh; (* This specifies the basal level of atoh
 transcription when Atoh protein and Hes protein are both absent. *)
pcritnregh = 1; (*defines the unit of pnicd*)
mcrithrega = 1; (*defines the unit of mhes*)
mcritarega = 1;
mcrithregd = 1;
mcritaregd = 1; (*defines the unit of matoh*)
mcritdregn = 1; (*defines the unit of mdelta*)
bmatoh = N[1/tmatoh];
bmhes = N[1/tmhes];
bmdelta = N[1/tmdelta];
bpnicd = N[1/tpnicd];
deltagenedosage = 1;

Atohfunc = 1; (*Set this to 0 to describe Atoh loss-of-function*)
Hesfunc = 1; (*Set this to 0 to describe Hes loss-of-function*)

f0[imhes, jcell_, t_, cisconcs_, rcisconcs_, rtransconcs_] :=
```

$$(1 - \text{bmhes})\ \text{cisconcs}[[\text{imhes}]] + \frac{\text{khes}\left(\frac{\text{rcisconcs}[[\text{ipnicd}]]}{\text{pcritnregh}}\right)^2}{1 + \left(\frac{\text{rcisconcs}[[\text{ipnicd}]]}{\text{pcritnregh}}\right)^2};$$

```
f0[imatoh, jcell_, t_, cisconcs_, rcisconcs_, rtransconcs_] :=
```

$$(1 - \text{bmatoh})\ \text{cisconcs}[[\text{imatoh}]] + \frac{\text{katoh}\left(\frac{\text{rcisconcs}[[\text{imatoh}]]}{\text{mcritarega}}\ \text{Atohfunc}\right)^2 + \text{katohExtra}}{\left(1 + \left(\frac{\text{rcisconcs}[[\text{imhes}]]}{\text{mcrithrega}}\ \text{Hesfunc}\right)^2 + \left(\frac{\text{rcisconcs}[[\text{imatoh}]]}{\text{mcritarega}}\ \text{Atohfunc}\right)^2\right)};$$

```
f0[imdelta, jcell_, t_, cisconcs_, rcisconcs_, rtransconcs_] :=
```

$$(1 - \text{bmdelta})\ \text{cisconcs}[[\text{imdelta}]] + \frac{\text{kdelta}\left(\frac{\text{rcisconcs}[[\text{imatoh}]]}{\text{mcritaregd}}\ \text{Atohfunc}\right)^2 * \text{deltagenedosage}}{\left(1 + \left(\frac{\text{rcisconcs}[[\text{imatoh}]]}{\text{mcritaregd}}\ \text{Atohfunc}\right)^2\right)};$$

```
f0[ipnicd, jcell_, t_, cisconcs_, rcisconcs_, rtransconcs_] :=
```

$$(1 - \text{bpnicd})\ \text{cisconcs}[[\text{ipnicd}]] + \frac{\text{kn}\left(\frac{\text{rtransconcs}[[\text{imdelta}]]}{\text{mcritdregn}}\right)}{1 + \left(\frac{\text{rtransconcs}[[\text{imdelta}]]}{\text{mcritdregn}}\right)};$$

Non-dimensionalization:

We can choose units for the protein and mRNA concentrations so as to make the critical concentrations equal to 1 (or any other value we please), for each of them, for any chosen one of its actions, leaving the other critical concentrations and the degradation rates b and the transcription initiation rates k and the cis- and trans-delays as the parameters to be explored.

■ Set the starting conditions and the dimensions of the tables of values that describe the system.

fullhistory is an array of values that describes the history of the system fully, specifying the concentration of each molecule at each time point in each cell. Specifically,
fullhistory [[t, jcell, imol]] is the concentration of molecule *imol* in cell *jcell* at timepoint *t*.
fullhistory [[t]] is a snapshot of the state of the system at timepoint *t*.

recenthistory is just that part of *fullhistory* that we need to know in order to compute the next state of the system.
recenthistory [[1]] is a snapshot of the state of the system at a time preceding the present by an amount *maxdelay*;
recenthistory [[maxdelay+1]] is a snapshot of the present state of the system; that is,
recenthistory [[maxdelay+1, jcell, imol]] is the present concentration of the molecule *imol* in cell *jcell*.

```
recenthistory0 =
  (SeedRandom[1]; Table[If [jm == imatoh, 1 * RandomReal[], 1 * RandomReal[]],
    {jt, 1, 1 + maxdelay}, {jcell, 1, ncells}, {jm, 1, nmols}]);
fullhistory0 = Table[If[jt > maxdelay + 1, 0, recenthistory0⟦jt, jcell, jm⟧],
   {jt, 1, tfinal}, {jcell, 1, ncells}, {jm, 1, nmols}];
```

- **Specify how to apply a full series of updates iteratively to obtain the full spatio-temporal history of the system as it develops subject to the chosen molecular controls, up to time tfinal.**

```
computebehaviour :=
  (
   fullhistory = fullhistory0;
   recenthistory = recenthistory0;

   timetocompute = Timing[
      Do[
       (
        currentCisMols = Table[
          recenthistory[[1 + maxdelay, jcell, mj]],
          {itargetmol, 1, nmols}, {jcell, 1, ncells}, {mj, 1, nmols}
         ];
        (* currentCisMols[[itargetmol,jcell,mj]]  is
         the current concentration of molecule #mj,  in cell #jcell,
        repeated identically  for all values of #itargetmol  *)
        retardedCisMols = Table[
          recenthistory[[1 + maxdelay - cisdelay[[itargetmol, mj]], jcell, mj]],
          {itargetmol, 1, nmols}, {jcell, 1, ncells}, {mj, 1, nmols}
         ];
        (* retardedCisMols[[itargetmol,jcell,mj]]
         is the concentration of molecule #mj,
        evaluated with the appropriate retardation for its current (timepoint t) cis-
         action on target molecule #itargetmol, in cell #jcell  *)
        retardedTransMols = Table[
          recenthistory[[1 + maxdelay - transdelay[[itargetmol, mj]], jcell, mj]],
          {itargetmol, 1, nmols}, {jcell, 1, ncells}, {mj, 1, nmols}
         ];
        (* retardedTransMols[[itargetmol,jcell,mj]]  is the concentration
         of molecule #mj, evaluated  in cell #jcell with the appropriate
          retardation for its current (timepoint t) trans-action on target
          molecule #itargetmol in the neighbours of cell #jcell . *)

        totNbrsRetardedTransMols = Table[
          Sum[retardedTransMols[[itargetmol, jnbr, mj]],
           {jnbr, neighbourindices[jcell]}],
          {itargetmol, 1, nmols}, {jcell, 1, ncells}, {mj, 1, nmols}
         ];
        (* totNbrsRetardedTransMols[[itargetmol,jcell,mj]] is the concentration of
         molecule #mj, evaluated with the appropriate retardation for its
          current (timepoint t) trans-action on target molecule #itargetmol,
        summed over all the neighbours of cell #jcell.  *)

        newstate = Table[
          f0[imol, jcell, t, currentCisMols[[imol, jcell]],
           retardedCisMols[[imol, jcell]], totNbrsRetardedTransMols[[imol, jcell]]],
          {jcell, 1, ncells} , {imol, 1, nmols}
         ];
        (*  newstate[[jcell,imol]]  is the concentration to be assigned
         to molecule #imol in cell #jcell at the next timepoint  *)

        recenthistory = Append[Drop[recenthistory, 1], newstate];

        fullhistory[[t + 1]] = newstate;
       ),
       {t, maxdelay + 1, tfinal - 1}
      ];
      allcells = Transpose[fullhistory, {3, 1, 2}];
     ][[1]];
  );
```

Specify how to display the results and graph the timecourse

```
printVals[listParameterNames_] :=
  Table[Print[listParameterNames[[jlistpn]] <> " = " , ToExpression[
     listParameterNames[[jlistpn]]]], {jlistpn, 1, Length[listParameterNames]}];
printCisDelayTable :=
 (cisDelayTable =
   Table[Flatten[{N[cisdelay[[im]] / minute], "  to control   " <> moltypes[[im]]}],
    {im, 1, nmols}];
  Print["\nDelay (in minutes) for controlling molecule in cis\n",
   Insert[cisDelayTable, moltypes, 1] // TableForm];)
printTransDelayTable := (transDelayTable =
   Table[Flatten[{N[transdelay[[im]] / minute], "  to control   " <> moltypes[[im]]}],
    {im, 1, nmols}];
  Print["\nDelay (in minutes) for controlling molecule in trans\n",
   Insert[transDelayTable, moltypes, 1] // TableForm];)
displaytimecourse :=
  (
   scaling = Table[1, {nmols}];
    (* Default - subsequent lines may modify *)
   scaling⟦imhes⟧ = mcrithrega;
   scaling⟦imatoh⟧ = mcritaregd;
   scaling⟦imdelta⟧ = mcritdregn;
   scaling⟦ipnicd⟧ = pcritnregh;
   scaledAllCells = Table[scaling[[jmol]] * fullhistory[[t, jcell, jmol]],
     {jcell, 1, ncells}, {jmol, 1, nmols}, {t, 1, tfinal}];
   graph[jcell_] := ListPlot[scaledAllCells[[jcell, {imhes, imatoh, imdelta, ipnicd},
       All]], Joined → {True, True, True, True}, PlotStyle →
       {{RGBColor[1, 0, 0], Thickness[0.002]}, {RGBColor[0, 1, 0], Thickness[0.002]},
        {RGBColor[0, 0, 1], Thickness[0.002]}, {RGBColor[1, 0, 1], Thickness[0.002]}},
     PlotRange → {{0, tfinal}, {0, 5}}, AspectRatio → 0.2, ImageSize → 80 * 8,
     PlotLabel → ("\n mhes (red), matoh (green), mdelta (blue),
          pnicd (purple), time in minutes\n  cell # " <>
        ToString[jcell] <> " at " <> ToString[addresses[[jcell]]]),
     Ticks → {Table[{100 * nt100, 100 * nt100 / minute}, {nt100, 0, tfinal / 100, 5}],
       Automatic}];
   gt = Table[graph[njcell], {njcell, 1, ncells}];
   (*    Show [Apply[GraphicsColumn,Print[gt]]];    *)
   Print["\nClick on graph and scroll sideways to see graphs for other cells"];
   Print[GraphicsRow[gt]];
  );
```

Specify how to display the honeycomb pattern of cells and its coloring

```
nucleardiam = .4;
membranethickness = 0.02;
intercellspace = 0.01;
colorscaling = Table[1, {nmols}];
(* Default scaling for colour display. Actual desired scaling set in next lines*);
colorscaling[[imhes]] = mcrithrega;
colorscaling[[imatoh]] = mcritaregd;
colorscaling[[imdelta]] = mcritdregn / 2;
colorscaling[[ipnicd]] = pcritnregh;
bkgrndcolor = {1, 1, 1} * 1;
cellColoring[t_] := (celljts = Table[allcells[[jcell, imol, t]] /
      (allcells[[jcell, imol, t]] + colorscaling[[imol]]), {imol, 1, nmols}];
   u = addresses[[jcell, 1]];
   v = addresses[[jcell, 2]];
   membranecolor = {1, 1, 1};
   cytoplasmcolor =
    {celljts[[imhes]], celljts[[imatoh]], celljts[[imdelta]]};
   nucleuscolor =
    {celljts[[ipnicd]], celljts[[ipnicd]], celljts[[ipnicd]]}
  );

centre[ni_, nj_] := ni * latticevector1 + nj * latticevector2;
hexverts = N[{{-Sqrt[3] / 2, 1 / 2}, {0, 1},
    {Sqrt[3] / 2, 1 / 2}, {Sqrt[3] / 2, -1 / 2}, {0, -1}, {-Sqrt[3] / 2, -1 / 2}}];
translate[vertexlist_, vector_] := Map[Plus[#, vector] &, vertexlist];
```

```
membrane[ni_, nj_] :=
  Polygon[translate[(1 - intercellspace) * hexverts, centre[ni, nj]]];
cytoplasm[ni_, nj_] := Polygon[
   translate[(1 - membranethickness - intercellspace) * hexverts, centre[ni, nj]]];
nucleus[ni_, nj_] := Disk[centre[ni, nj], {nucleardiam, nucleardiam}];
cell[ni_, nj_, membranecolor_, cytoplasmcolor_, nucleuscolor_] := Graphics[{
    RGBColor[membranecolor], membrane[ni, nj],
    RGBColor[cytoplasmcolor], cytoplasm[ni, nj],
    RGBColor[nucleuscolor], nucleus[ni, nj]
   }];


displaySimple[t_] :=
  Show[
   Table[
    (cellColoring[t];
     cell[u, v, membranecolor, cytoplasmcolor, nucleuscolor]
    ),
    {jcell, 1, ncells}
   ],
   Background → Apply[RGBColor, bkgrndcolor],
   (*PlotRange→{{leftmargin,rightmargin},{bottommargin,topmargin}},*)
   AspectRatio → Automatic, PlotLabel → Null,
   ImageSize → 30 * {n1, n2}
  ];

displayCyclic[t_] :=
 (horizrepetition = 1;
  vertrepetition = 1;
  jhoriz = Ceiling[horizrepetition + n2 / 2];
  jvert = vertrepetition;
  leftmargin = Norm[latticevector1] * (1 + n1 * n2 / 2);
  rightmargin = Norm[latticevector1] * ((1 + jhoriz) * n1 - 1);
  bottommargin = Norm[latticevector1] * N[Sqrt[3] / 2];
  topmargin = Norm[latticevector1] * N[Sqrt[3] / 2] * ((1 + jvert) * n2 - 1);
  Show[
   Table[
    (cellColoring[t];
     Table[
      cell[u + n1 * jn1, v + n2 * jn2, membranecolor, cytoplasmcolor, nucleuscolor],
      {jn1, 0, jhoriz}, {jn2, 0, jvert}
     ]
    ),
    {jcell, 1, ncells}
   ],
   Background → Apply[RGBColor, bkgrndcolor], PlotRange →
    {{leftmargin, rightmargin}, {bottommargin, topmargin}}, AspectRatio → Automatic,
   PlotRangeClipping → True,
   PlotLabel → timelabel,
   ImageSize → 30 * {n1 * horizrepetition, n2 * vertrepetition}
  ]
 )
```

- **Specify movie**

```
makemovie :=
  (
   tstartshow = 1 ;
   nframes = 3;
   tinterval = Floor[tfinal / nframes] - 1;
   Do[
    (timelabel = Style["t = " <> ToString[0.1 * Round[10 * tf / (60 minute)]] <> " hours",
       "Section", FontSize → 14];

     Print[displaySimple[tf]];

     Print[displayCyclic[tf]];

    ),
    {tf, tstartshow, tfinal, tinterval}];

  );
```

Do the computation and display results

Values specified here for rate constants, critical concentrations and other parameters appearing in the dynamical equations override default values specified earlier

```
deltavals = {4, 1, .25};
printVals[{"moltypes", "minute/timestep", "tfinal", "ncells",
   "pcritnregh", "mcrithrega", "mcritarega", "mcritaregd", "mcritdregn",
   "tmatoh", "tmhes", "tmdelta", "tpnicd", "deltagenedosage", "khes*tmhes",
   "katoh*tmatoh", "kdelta*tmdelta", "kn*tpnicd", "katohExtra*tmatoh"}];
printCisDelayTable;
printTransDelayTable;
Print["\n\n————————————————————————\nValues respecified
    below override defaults printed above\n————————————————————————"];
Do[
 (
  deltagenedosage = deltavals[[ideltavals]];
  Print["\n\n"];
  printVals[{"deltagenedosage"}];
  computebehaviour;
  printVals[{"timetocompute"}];
  displaytimecourse;
  makemovie;
  Print["moltypes = ", moltypes];
  Print["Final state (as list of cell states)= \n", allcells[[All, All, tfinal]]];
  Print["BinCount of numbers of cells at \ndifferent final levels of atoh mRNA = \n",
   BinCounts[allcells[[All, imatoh, tfinal]], .5]];
  Print["Histogram of numbers of cells at \ndifferent final levels of atoh mRNA: \n",
   Histogram[allcells[[All, imatoh, tfinal]], 50, ImageSize → 200]];),
 {ideltavals, 1, Length[deltavals]}
]
```

```
moltypes = {matoh, mdelta, mhes, pnicd}

minute/timestep = 1

tfinal = 4000

ncells = 20

pcritnregh = 1

mcrithrega = 1

mcritarega = 1

mcritaregd = 1

mcritdregn = 1

tmatoh = 200

tmhes = 10

tmdelta = 10
```

```
tpnicd = 10

deltagenedosage = 1

khes*tmhes = 5.

katoh*tmatoh = 5.

kdelta*tmdelta = 0.2

kn*tpnicd = 5.

katohExtra*tmatoh = 0.5

Delay (in minutes) for controlling molecule in cis

matoh    mdelta    mhes    pnicd
22.      0.        32.     0.          to control    matoh
22.      0.        32.     0.          to control    mdelta
0.       0.        0.      30.         to control    mhes
0.       0.        0.      0.          to control    pnicd

Delay (in minutes) for controlling molecule in trans

matoh    mdelta    mhes    pnicd
0.       0.        0.      0.          to control    matoh
0.       0.        0.      0.          to control    mdelta
0.       0.        0.      0.          to control    mhes
0.       40.       0.      0.          to control    pnicd

______________________
Values respecified below override defaults printed above
______________________

deltagenedosage = 4

timetocompute = 24.8063

Click on graph and scroll sideways to see graphs for other cells
```

mhes (red), matoh (green), mdelta (blue), pnicd (purple), time in minutes
cell ⌗ 1 at {0, 0}

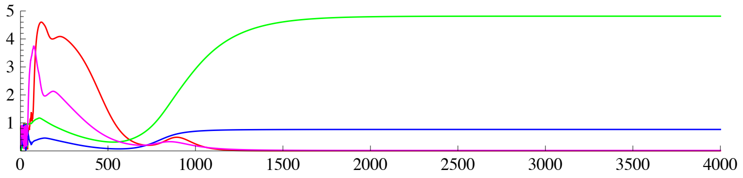

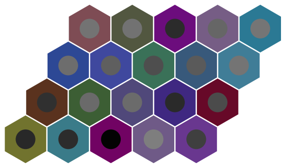

t = 0 hours

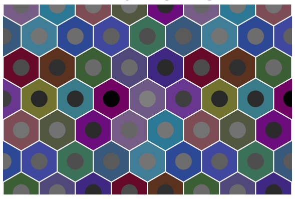

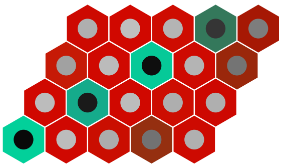

t = 22.2 hours

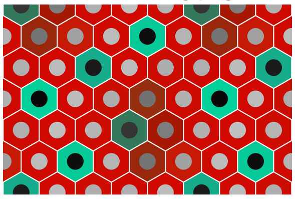

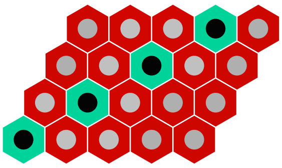

t = 44.4 hours

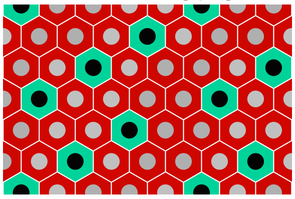

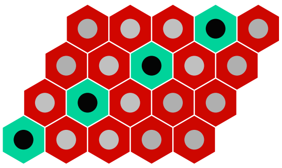

t = 66.6 hours

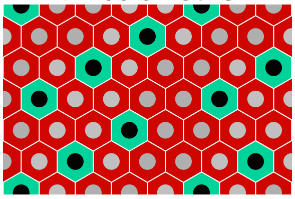

```
moltypes = {matoh, mdelta, mhes, pnicd}
```

```
Final state (as list of cell states)=
{{4.81384, 0.766905, 0.00127562, 0.0159747},
  {0.0235752, 0.000444386, 4.50844, 3.02849},
  {0.0279408, 0.000624063, 4.12723, 2.17461},
  {0.0279405, 0.000624052, 4.12724, 2.17461},
  {0.0235765, 0.000444433, 4.50832, 3.02807},
  {4.81383, 0.766905, 0.00100661, 0.0141902},
  {0.0235756, 0.000444402, 4.5084, 3.02835}, {0.023576, 0.000444417, 4.50836, 3.02821},
  {0.0235761, 0.00044442, 4.50836, 3.02821}, {0.0235756, 0.000444402, 4.5084, 3.02835},
  {4.81384, 0.766905, 0.0010066, 0.0141901}, {0.0235765, 0.000444435, 4.50832, 3.02807},
  {0.0279414, 0.000624095, 4.12723, 2.17461},
  {0.0279406, 0.000624054, 4.12724, 2.17461},
  {0.0235753, 0.000444387, 4.50844, 3.02849},
  {4.81381, 0.766905, 0.00127575, 0.0159753},
  {0.0279356, 0.000623832, 4.12762, 2.17518},
  {0.0279405, 0.000624052, 4.12724, 2.17461},
  {0.0279412, 0.000624086, 4.12723, 2.17461}, {0.027936, 0.00062385, 4.12761, 2.17518}}

BinCount of numbers of cells at
different final levels of atoh mRNA =
{16, 0, 0, 0, 0, 0, 0, 0, 0, 4}

Histogram of numbers of cells at
different final levels of atoh mRNA:
```

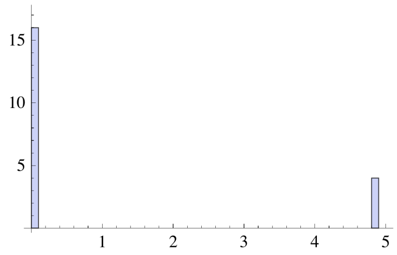


```
deltagenedosage = 1

timetocompute = 24.9823

Click on graph and scroll sideways to see graphs for other cells
```

mhes (red), matoh (green), mdelta (blue), pnicd (purple), time in minutes
cell ⌗ 1 at {0, 0}

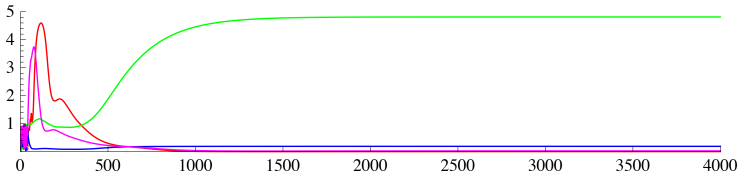

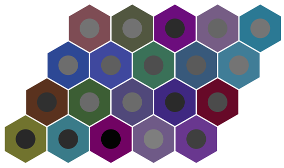

t = 0 hours

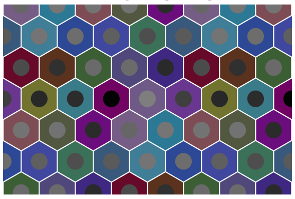

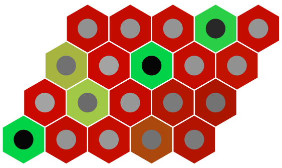

t = 22.2 hours

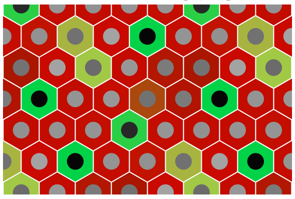

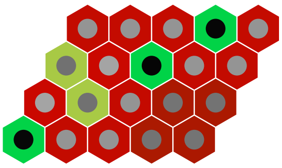

t = 44.4 hours

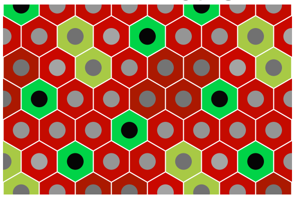

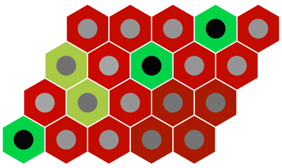

t = 66.6 hours

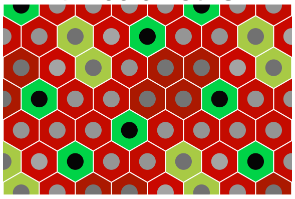

```
moltypes = {matoh, mdelta, mhes, pnicd}
```

```
Final state (as list of cell states) =

 {{4.81384, 0.191726, 0.00443976, 0.029812}, {0.0321846, 0.000206957, 3.83348, 1.81281},
  {3.7814, 0.186925, 1.93068, 0.793101}, {0.0432868, 0.000374062, 3.28128, 1.38173},
  {0.0434768, 0.000377327, 3.27341, 1.3769}, {3.79065, 0.186988, 1.92989, 0.79286},
  {0.0322502, 0.000207801, 3.8294, 1.80868}, {0.0429737, 0.000368667, 3.29364, 1.38932},
  {0.0432412, 0.000373255, 3.28276, 1.38262},
  {0.0430562, 0.000370074, 3.29019, 1.38719},
  {4.81384, 0.191726, 0.00194253, 0.0197149},
  {0.0429578, 0.000368395, 3.29428, 1.38972}, {0.10979, 0.00238221, 2.02286, 0.824298},
  {0.10744, 0.00228227, 2.04425, 0.831636}, {0.0425537, 0.000361509, 3.31072, 1.39994},
  {4.81384, 0.191726, 0.00221668, 0.0210589}, {0.107424, 0.00228159, 2.04439, 0.831683},
  {0.109965, 0.00238959, 2.02112, 0.823701}, {0.0432758, 0.000373872, 3.28171, 1.38199},
  {0.0430543, 0.000370059, 3.29057, 1.38744}}

BinCount of numbers of cells at
different final levels of atoh mRNA =
{15, 0, 0, 0, 0, 0, 0, 2, 0, 3}

Histogram of numbers of cells at
different final levels of atoh mRNA:
```

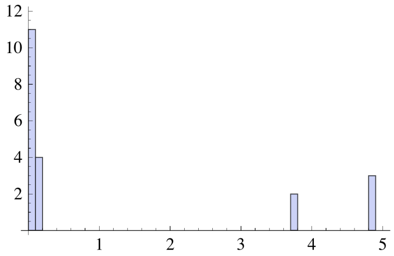


```
deltagenedosage = 0.25

timetocompute = 25.149

Click on graph and scroll sideways to see graphs for other cells
```

mhes (red), matoh (green), mdelta (blue), pnicd (purple), time in minutes
cell # 1 at {0, 0}

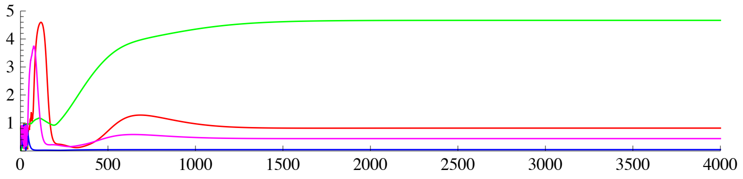

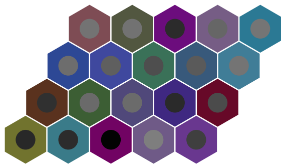

t = 0 hours

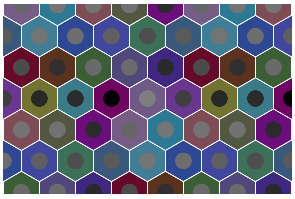

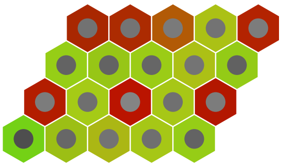

t = 22.2 hours

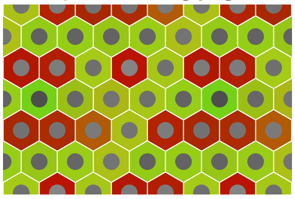

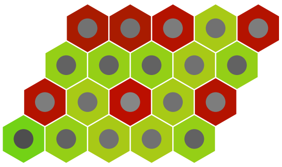

t = 44.4 hours

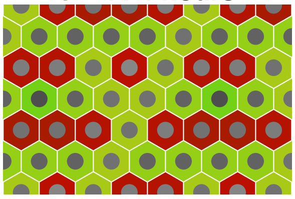

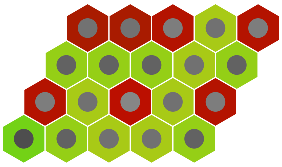

t = 66.6 hours

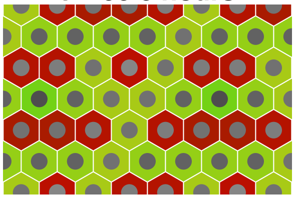

```
moltypes = {matoh, mdelta, mhes, pnicd}

Final state (as list of cell states)=
{{4.66564, 0.047804, 0.816797, 0.441878}, {0.0787517, 0.00030818, 2.39512, 0.95889},
  {4.3407, 0.04748, 1.406, 0.625464}, {0.11721, 0.00067761, 1.95916, 0.802671},
  {4.34462, 0.0474844, 1.40079, 0.623855}, {3.7693, 0.0467123, 1.94282, 0.79718},
  {4.33834, 0.0474774, 1.40902, 0.626398}, {0.117191, 0.000677389, 1.95929, 0.802717},
  {3.78821, 0.0467423, 1.92711, 0.791917}, {0.0610754, 0.000185819, 2.73651, 1.09954},
  {4.34891, 0.0474891, 1.39519, 0.622124}, {0.0795477, 0.000314424, 2.38294, 0.954226},
  {3.79212, 0.0467491, 1.92716, 0.791936}, {3.77991, 0.0467294, 1.93506, 0.794579},
  {3.77572, 0.0467224, 1.93651, 0.795065}, {3.78853, 0.0467432, 1.92859, 0.792414},
  {4.34441, 0.0474841, 1.4011, 0.623949}, {0.0791585, 0.000311356, 2.38877, 0.956456},
  {4.35114, 0.0474915, 1.39233, 0.621239}, {0.0791099, 0.000310975, 2.38951, 0.956739}}
```

BinCount of numbers of cells at different final levels of atoh mRNA = {7, 0, 0, 0, 0, 0, 0, 6, 6, 1}

Histogram of numbers of cells at different final levels of atoh mRNA:

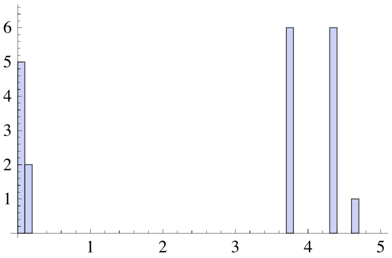

Supplement: Text S2 — A model of Delta-Notch-mediated lateral inhibition in a sheet of intestinal epithelial cells: Effects of varying Delta gene dosage. This Mathematica program computes the behavior of an array of cells interacting with one another via the Delta-Notch lateral-inhibition pathway as indicated by the gene-regulatory circuit diagram shown in Figure 7. The cells are assumed to start off all in a similar state, but with some minor random variation from one cell to the next. The computation shows that the pattern of cell states that ultimately emerges depends on the number of functional Delta gene copies that the cells contain, in the manner summarised in Figure 7. (PDF) [file pone.0024484.s002.pdf]
